# Automated Fiber Diameter and Porosity Measurements of Plasma Clots in Scanning Electron Microscopy Images

**DOI:** 10.3390/biom11101536

**Published:** 2021-10-18

**Authors:** Ali Daraei, Marlien Pieters, Stephen R. Baker, Zelda de Lange-Loots, Aleksander Siniarski, Rustem I. Litvinov, Caroline S. B. Veen, Moniek P. M. de Maat, John W. Weisel, Robert A. S. Ariëns, Martin Guthold

**Affiliations:** 1 Department of Physics, Wake Forest University, Winston-Salem, NC 27109, USA; alidaraei1361@ucla.edu (A.D.); bakersr@wfu.edu (S.R.B.); 2 Center of Excellence for Nutrition (CEN), Potchefstroom Campus, North-West University, Potchefstroom 2520, South Africa; Zelda.DeLange@nwu.ac.za; 3 Medical Research Council Unit for Hypertension and Cardiovascular Disease, Potchefstroom Campus, North-West University, Potchefstroom 2520, South Africa; 4 Leeds Institute of Cardiovascular and Metabolic Medicine, University of Leeds, Leeds LS16 8FX, UK; r.a.s.ariens@leeds.ac.uk; 5 Department of Coronary Disease and Heart Failure, Institute of Cardiology, Jagiellonian University Medical College, 31-202 Krakow, Poland; aleksandersiniarski@gmail.com; 6 John Paul II Hospital, 31-202 Krakow, Poland; 7 Department of Cell and Developmental Biology, Perelman School of Medicine, University of Pennsylvania, Philadelphia, PA 19104, USA; litvinov@pennmedicine.upenn.edu (R.I.L.); weisel@pennmedicine.upenn.edu (J.W.W.); 8 Department of Hematology, Erasmus University Medical Center, 3015 GD Rotterdam, The Netherlands; c.s.b.veen@erasmusmc.nl (C.S.B.V.); m.demaat@erasmusmc.nl (M.P.M.d.M.)

**Keywords:** automated analysis, DiameterJ, structure, fibrin fibers, plasma clots, diameter, porosity, scanning electron microscopy

## Abstract

Scanning Electron Microscopy (SEM) is a powerful, high-resolution imaging technique widely used to analyze the structure of fibrin networks. Currently, structural features, such as fiber diameter, length, density, and porosity, are mostly analyzed manually, which is tedious and may introduce user bias. A reliable, automated structural image analysis method would mitigate these drawbacks. We evaluated the performance of DiameterJ (an ImageJ plug-in) for analyzing fibrin fiber diameter by comparing automated DiameterJ outputs with manual diameter measurements in four SEM data sets with different imaging parameters. We also investigated correlations between biophysical fibrin clot properties and diameter, and between clot permeability and DiameterJ-determined clot porosity. Several of the 24 DiameterJ algorithms returned diameter values that highly correlated with and closely matched the values of the manual measurements. However, optimal performance was dependent on the pixel size of the images—best results were obtained for images with a pixel size of 8–10 nm (13–16 pixels/fiber). Larger or smaller pixels resulted in an over- or underestimation of diameter values, respectively. The correlation between clot permeability and DiameterJ-determined clot porosity was modest, likely because it is difficult to establish the correct image depth of field in this analysis. In conclusion, several DiameterJ algorithms (M6, M5, T3) perform well for diameter determination from SEM images, given the appropriate imaging conditions (13–16 pixels/fiber). Determining fibrin clot porosity via DiameterJ is challenging.

## 1. Introduction

Scanning electron microscopy (SEM) images of fibrin networks provide critical information on the structure of clots, such as fiber orientation, branching, diameter, length, fiber density, and network pore size or porosity [1,2,3,4,5,6,7,8,9]. These microscopic characteristics are related to macroscopic clot properties, such as permeability, lytic resistance, or viscoelasticity, which, in turn, have been shown to associate with disease. For example, clots with thinner, more densely packed fibers, and smaller pores and more branch points result in a stiffer network, reduced permeability, and increased resistance to fibrinolysis [10,11,12]. Conversely, clots formed from thicker fibers generally have looser and less stiff networks that are more permeable and more susceptible to fibrinolysis [10,13,14,15]. Plasma clots with increased mechanical stiffness and increased resistance to fibrinolysis have been consistently related to a variety of arterial and venous thrombotic diseases [16,17,18,19,20,21,22,23]. Clot structure and stability may also be used to assess prevalence and treatment of bleeding disorders, such as hemophilia A and B [20,22]. 

SEM images of fibrous networks are typically analyzed manually; i.e., a user inspects the images and makes measurements by hand [24,25,26]. This process is tedious, may introduce user bias, and typically limits the sample size for which this type of analysis is considered. A reliable, automated analysis method of fibrous networks in SEM images would mitigate some of these problems and facilitate faster image analysis. An automated analysis method would also be useful in the development of standardization protocols to investigate the structure of blood clots in hemostasis and thrombosis. There are several segmentation algorithms to recognize and statistically analyze fiber diameters. However, automated image analysis programs for fiber measurements are still used infrequently due to the lack of reliable and widely available software to quantify network properties from SEM images of fibrous meshes [2]. 

Recently, DiameterJ, a plug-in for the open-source, NIH-supported image analysis program ImageJ, was introduced. This plug-in was developed to analyze SEM images of porous nanofiber scaffolds [27]. The developers tested it on three sets of SEM images to validate the program. The image sets were synthetic, digital images of fibers with known diameters; steel wires with known diameters; and electrospun polymeric nanofibers with unknown diameters [28]. In 2017, the applicability of DiameterJ analysis for determining the diameter of fibrin fibers in SEM images was briefly assessed, with the conclusion that more work is needed [29]. DiameterJ provides 24 algorithms that automatically segment gray-scale SEM images into binary black (background) and white (object/fiber) images. The segmented images are then used to automatically determine the distribution of fiber diameters and other structural features (e.g., porosity). More details pertaining to the algorithms and segmentation are provided in the Online Supplement.

The overall aim of our study was to evaluate which of the 24 DiameterJ algorithms are well suited to reliably analyze and quantify the microstructure of fibrin meshes in SEM images. The specific objectives of the study were to: (1A) Evaluate the accuracy and utility of the 24 segmentation algorithms included in DiameterJ on three large data sets of 120 SEM images of plasma clots (set 1, 10 postpartum hemorrhage and 5 healthy participants (control participants)), 69 SEM images (set 2, 23 patients with acute myocardial infarction (AMI)) and 150 SEM images (set 3, 30 healthy participants) by comparing the outcomes to manual diameter measurements. (1B) Evaluate the emerging best subset of the 24 algorithms on two patient data sets to determine if trends in samples from healthy individuals and individuals with diseases can be reliably detected (sets 1 and 4). (2) Evaluate which segmentation algorithm best represents clot structure and thus may be used for porosity measurements. (3) Evaluate how well the automated and manual diameter and porosity measurements correlate with clot properties determined by complementary techniques (fibrinogen concentration, turbidity, permeability, and viscoelasticity) in 150 SEM images of plasma samples from 30 participants. 

Our data indicate that the diameter values returned by DiameterJ depend on the pixel size of the images, with larger pixels returning larger diameter values. However, when using appropriate imaging conditions, several DiameterJ algorithms return accurate diameter values, and these conditions can be recommended for fibrin fiber diameter analysis. Porosity analysis using SEM images is challenging since it is difficult to determine the correct image depth of field. Automated and manual diameter measurements correlated similarly with several clot properties. 

## 2. Materials and Methods

The SEM datasets were obtained from four different labs, which performed SEM analyses on plasma clots using their “in-house” methods for sample preparation and imaging, and a standard method of fiber diameter measurement, as part of studies investigating clot structure. These standard methods for diameter measurement all involve randomly selecting 54–100 fibers per image and manually determining the diameter of these fibers using ImageJ. We selected multiple datasets to determine if the automated analysis can be successfully applied to images obtained in different labs, and, thus, demonstrate broad applicability. 

### 2.1. Set 1: Postpartum Hemorrhage (PPH) Samples

#### 2.1.1. Patient Population and Study Design

As part of the Crescendo study, patients with Bleeding of Unknown Cause (BUC) and healthy controls were recruited from July 2016 until March 2018 at the outpatient Haematology Clinic of the Erasmus University Medical Center, Rotterdam, the Netherlands. For this pilot study, 10 women with severe PPH (patients) and 5 women with a normal delivery (controls) were selected (Table 1). Severe PPH was defined as blood loss larger than 2000 mL within 24 h after delivery. This study was subject to the Medical Research Involving Human Subjects Act and approved by the Medical Ethics Committee of the Erasmus University Medical Center Rotterdam (MEC-2016-218, 2016). All participants gave written informed consent. 

#### 2.1.2. Plasma Preparation and Clinical Coagulation Testing

Blood samples were acquired at least three months after delivery. Blood sampling was performed by venipuncture using the Vacutainer system (Becton Dickinson, Utrecht, The Netherlands) containing 3.2% sodium citrate. Citrated blood was centrifuged two times at 2000× *g* for 10 min at room temperature, followed by 14,000× *g* for 10 min centrifugation of plasma at room temperature. Plasma samples were stored in aliquots at −80 °C until analysis. Fibrinogen activity according to the Clauss method (Thrombin Reagent, Siemens Healthcare Diagnostics B.V., Breda, The Netherlands) was measured on a Sysmex CS5100 (Siemens Healthcare Diagnostics B.V.). 

#### 2.1.3. Scanning Electron Microscopy

Clots for SEM were prepared as described previously [30]. Briefly, clot formation was initiated by the addition of thrombin and CaCl_2_ to platelet-poor plasma, with a final concentration of 0.5 U/mL Thrombin and 25 mM CaCl_2_. Clots were left to form at room temperature in a dark and moist atmosphere for 30 min. After polymerization, clots were washed three times with sodium cacodylate buffer and subsequently fixed in a 2% glutaraldehyde solution for 2 h. The fixed clots were then stepwise dehydrated in 30, 50, 70, 90, and 95% ethanol solutions, and 3 times in 100% ethanol. The procedure was completed by drying with hexamethyldisilazane (HMDS), and sputter coating with gold/palladium. The clots were examined and photographed with a Quanta FEG 250 FEI/Thermo Fisher Scientific scanning electron microscope (Hillsboro, OR, USA). In total, 8 images of every clot in randomly selected areas of the clot were acquired at 10,000× magnification.

### 2.2. Set 2: Acute Myocardial Infarction (AMI) Samples

#### 2.2.1. Patient Population and Study Design 

Twenty-three patients who were hospitalized within 12 h of AMI symptoms onset were included in the original study [31]. This study included patients with ST-segment elevation MI (STEMI) and non-ST-segment elevation MI (NSTEMI) whose diagnoses were established according to the Third Universal Definition of Myocardial Infarction [32]. This study complied with the Declaration of Helsinki and was approved by the Ethics Committee of Jagiellonian University (KBET/122.6120.271.2015) and the University of Leeds Research Ethics Committee (HSLTLM12045, 2016). Each participant provided written informed consent.

#### 2.2.2. Plasma Preparation

Upon admission to the Emergency Department, blood samples were collected from the antecubital vein with minimal stasis. Citrated blood was centrifuged within 15–60 min after collection. After centrifugation, plasma was collected and stored at −70 °C until further analysis. Total fibrinogen levels were determined using the Clauss method with a Start 4 hemostasis analyzer (Diagnostica Stago, Theale, UK) and Fibri-prest automate kit (Diagnostica Stago, Theale, UK) according to manufacturer’s instructions. All measurements were done in duplicate. 

#### 2.2.3. Scanning Electron Microscopy

Clots for SEM were prepared by adding 1 volume of activation mix (10 mM CaCl_2_, 0.5 U/mL human α-thrombin, final concentrations) to 10 volumes of plasma, mixed, and the mixture was transferred to a pierced lid of a 0.6 mL Eppendorf tube (Eppendorf UK Limited, Stevenage, UK), as described in [31]. Clots were allowed to form in the lid for 2 h inside a humid chamber at room temperature. Once clots were fully formed, they were washed in saline (0.9 g/L) for at least 2 h and then fixed in 2% glutaraldehyde solution overnight. The following day, clots were washed in sodium cacodylate buffer (67 mM C_2_H_6_AsNaO_2_, pH 7.4) for at least 2 h followed by dehydration using a series of increasing acetone concentrations (30–100%). Fully fixed and dehydrated clots were critical point dried with CO_2_, mounted onto SEM stubs, and sputter-coated with iridium using a Cressington 208 HR (Cressington Scientific Instruments, Watford, UK). Images of clots were taken in 3 areas, at 3 different magnifications (5000×, 10,000×, and 20,000×) using a Hitachi SU8230 high-performance cold emission SEM equipped with an Oxford Instruments Aztec Energy EDX system with 80 mm X-Max SDD detector (Chiyoda, Tokyo, Japan). 

### 2.3. Set 3: Healthy Samples

#### 2.3.1. Population and Study Design

Data for this study were obtained from apparently healthy, Black South Africans (>30 years old) participating in the South African arm of the International Prospective Urban and Rural Epidemiology (PURE) study [33]. For the current study, a sub-sample of 30 participants was systematically selected from the larger study population with total fibrinogen concentrations ranging from 2.4 to 6.4 g/L. All PURE participants gave voluntary written informed consent and data collection complied with the Declaration of Helsinki of 1975 (as revised in 2013). The study was approved by the Health Research Ethics Committee of North-West University, South Africa (NWU-00016-10-A1, 2010). Fasting blood samples were collected between 07:00 a.m. and 11:00 a.m. from the antecubital vein using sterile winged infusion sets and syringes into 3.2% sodium citrate tubes. Samples were centrifuged within 30 min of collection at 2000× *g* for 15 min and the plasma was stored at −80 °C until further analysis [34].

#### 2.3.2. Turbidity

A modified turbidity assay [35] was used to determine formation, structure, and fibrinolytic potential of plasma clots. Final concentrations were 80 ng/mL tissue plasminogen activator (tPA; Actilyse, Boehringer Ingelheim, Ingelheim, Germany), 17 mM CaCl_2_, 1750× diluted tissue factor (TF; Dade Innovin, Siemens Healthcare Diagnostics Inc., Marburg, Germany) and 10 mM phospholipid vesicles (Rossix, Mölndal, Sweden). The TF and tPA concentrations were selected to obtain clot lysis times (CLTs) between 60 and 100 min in control plasma. Sigmoidal curve fitting [Origin^®^ software version 8.5 (Origin lab^®^, 2010)] was used to determine clot lysis time (CLT). Lag time (minutes), slope (×10^−3^ au/s), and maximum absorbance (Δau) were calculated as previously described [34].

#### 2.3.3. Rheometry

Viscoelastic properties of plasma clots (1 U/mL human α-thrombin and 20 mM CaCl_2_) were measured through oscillatory shear measurements at 37 °C on an ARES-G2 Rheometer (TA Instruments, New Castle, DE, USA) during plasma clot formation, as described in [34]. Time-sweep tests, using a 40 mm stainless steel cone, under an oscillation procedure of 3% strain, at an angular frequency of 5 radians per second (rad/s) (10 half sampling cycles) and with a sampling interval of 3 points per second were performed. Immersion oil (viscosity, 100–120 mPa·s) (Merck, Darmstadt, Germany) was placed around the cone to prevent drying of the outside edges of the clot. Storage modulus (G’) and loss modulus (G’), indicating elastic and viscous properties, respectively, were measured at 3 s intervals for 40 min for each plasma sample. 

#### 2.3.4. Permeability

Clot permeability (*K_s_*), an indication of the intrinsic pore size of a fibrin network, was measured as described previously in [34,36]. Plasma clots (1 U/mL human α-thrombin, Merck, Darmstadt, Germany, and 20 mM CaCl_2_) were prepared in triplicate in 3 cm sections of 1 mL plastic serological pipettes. Buffer was permeated through at a pressure height of *Δh* = 4 cm and *K_s_* was calculated from $K_{s}=\frac{\Delta Q\times L\times \eta }{\Delta T\;\times A\;\times \Delta P}$.

*ΔQ/ΔT* is the flow rate (flow-through volume/time), η is the dynamic viscosity (*η_water_* = 1.0 cP at 20 °C), *L* is the clot length, *A* is the cross-sectional area of the clot, and ΔP is the pressure drop. *ΔP* = *ρ_water_* × *g* × *Δh*, where the density of water, *ρ_water_* = 1 g/cm^3^, the acceleration due to gravity, *g* = 980 cm/s^2^, and *Δh* = 4 cm is the height from the meniscus of the buffer to the bottom of the clot.

#### 2.3.5. Scanning Electron Microscopy

Plasma clots used in the permeability measurements described above were rinsed with a cacodylate buffer and fixed in 2% glutaraldehyde (Merck, Darmstadt, Germany) overnight. Samples were then subjected to increasing doses of ethanol to enable dehydration and dried using HMDS (Merck, Darmstadt, Germany). These prepared clots were mounted onto SEM stubs and sputter-coated with gold/palladium before being viewed and photographed using an FEI Corporation Quanta 200 ESEM (Hillsboro, OR, USA) at 12,000× magnification and saved as TIF images. Five micrographs were obtained for each plasma sample. The coefficient of variation for all methods was <10%. 

### 2.4. Set 4: Systemic Lupus Erythematosus (SLE) Samples

#### 2.4.1. Patient Population and Study Design

The data for set 4 came from a study on the effect of systemic lupus erythematosus (SLE) on fibrin properties [24]. Twenty-eight patients, with 82/18% female/male ratio at a mean age of 37 ± 13 years, were included in the original study, based on the revised criteria of the American College of Rheumatology for SLE. Informed consent was obtained from the SLE patients and healthy donors under the approval of the Ethical Committee of the Kazan State Medical University (Kazan, Russia; protocol #2 as of 21 February 2017). A subset of all available SEM images from this study was included in the current study. Blood was drawn under aseptic conditions without venous stasis using vacutainers containing 3.8% trisodium citrate (Greiner Bio-One, Kremsmünster, Austria) at 9:1 *v*/*v*. For investigations of plasma clots, the blood was centrifuged at room temperature for 15 min at 1500× *g* to obtain platelet-poor plasma, then 5 min at 10,000× *g* to obtain platelet-free plasma, which was then aliquoted and stored at −80 °C until further analysis. For control experiments, platelet-free plasma from at least 10 healthy donors was pooled, aliquoted, and stored at −80 °C until use. The frozen samples were thawed at 37 °C for 60 min and used within 2 h.

#### 2.4.2. Scanning Electron Microscopy

Fibrin clots, formed by adding CaCl_2_ (24 mM final concentration) without thrombin to platelet-free plasma, were allowed to form for 2 h at room temperature in a humid chamber. The clots were washed in 0.05 M sodium cacodylate buffer (pH 7.4), then fixed in 2% glutaraldehyde in the same buffer, dehydrated in ethanol, dried with HMDS, and sputter-coated with gold/palladium. The samples were imaged with a Merlin scanning electron microscope (Carl Zeiss, Oberkochen, Germany). Fiber thickness was measured using ImageJ (remeasurement of the original data sets) at randomly selected areas for 100 fibers per clot. 

### 2.5. Fiber Diameter Measurement

#### 2.5.1. Manual Measurement of Fiber Diameter

**Figure 1:**
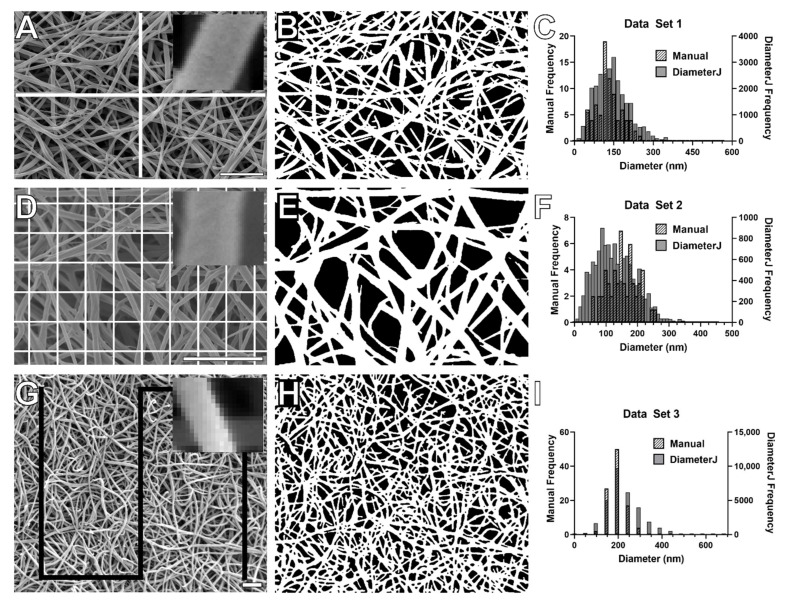
Manual and DiameterJ analysis of three large data sets. SEM images and patterns for manual analysis are shown for data set 1 (**A**), set 2 (**D**), and set 3 (**G**). The line pattern in each image was used to select 100, 54, and 100 random fibers per image for manual diameter measurements. Representative DiameterJ binary images are shown for set 1 (**B**), set 2 (**E**), and set 3 (**H**). Frequency distributions for manual and DiameterJ diameter measurements are shown for data set 1 (**C**), set 2 (**F**), and set 3 (**I**). The insets in (**A**,**D**,**G**) show the varying pixel size across sets; 8.3 nm in set 1, 4.9 nm in set 2 and 24.3 nm in set 3. Scale bars in (**A**,**D**,**G**) are 2 μm.

The line tool in ImageJ (v 1.48, NIH, Bethesda, MD, USA) was used to manually measure the fiber diameters in all data sets [37,38]. The diameters of fibrin fibers were measured using three, slightly different, but equivalent standard manual methods. In set 1 and set 4, the diameters of 100 random fibers that transected a cross-shaped pattern superimposed on the SEM images were measured (Figure 1A). In set 2, the diameters of the 54 fibers that were closest to the 54 line crossing points were measured (Figure 1D). In set 3, the diameters of 100 random fibers that intersected a rectangular line pattern were measured (Figure 1G). Care was taken to measure a fiber only once. This is the standard procedure currently used to determine fiber diameter from SEM images [24,39,40,41]. 

#### 2.5.2. DiameterJ-Based Fiber Diameter Measurements

Automated fiber diameter measurements were investigated using DiameterJ. Based on the different segmentation algorithms, the SEM images were first converted into black and white (i.e., binary) representations of the images, in which the white pixels represent fibers and the black pixels represent the background (Figure 1B,E,H). The DiameterJ plugin offers 24 different segmentation algorithms, which are classified into three categories: (1) Traditional (T1–T8), (2) Mixed (M1–M8), and (3) Statistical Region Merging (S1–S8), each containing 8 different segmentation algorithms. Details of the segmentation algorithms and techniques implemented in DiameterJ are described in the Supplementary Materials; also, see [27].

DiameterJ uses three different averaging approaches to determine the mean fiber diameter from the diameter distribution for a given image: (1) The simple arithmetic mean of the individual diameter measurements; (2) super pixel algorithm, which yields the most accurate results for multimodal distributions; and (3) histogram mean, which is the peak of a Gaussian fit to the distribution of diameters. 

### 2.6. DiameterJ-Based Network Porosity Measurements

To calculate the mean pore area and the percent porosity of fibrin networks, the segmentation that visually best represented the original SEM images (segmented images that closely match the original SEM images) was chosen. In DiameterJ, the pore space refers to the area of the image that is not segmented as fiber [27] and is expressed as percent porosity. After visual inspection of SEM images that were overlaid with the segmented images, we determined that segmentation algorithms T5 and T6 provided the closest versions to the original SEM images. Accordingly, these algorithms were utilized to calculate the mean pore area and the percent porosity of all five images of each of the thirty plasma clots from participants of Set 3 (150 total images). Set 3 was used for the porosity measures because it was the only set with experimentally available permeability data.

## 3. Results

An overarching observation in our evaluation was that the 24 algorithms can be ranked by the relative magnitude of their diameter output values (Table 2). Specifically, for all data sets, algorithms S4, S8, T4, and M8 (group 1) generally returned significantly below average diameter values (below one standard deviation σ compared to the mean of all algorithms for a given set, σ = 8.2 for the deviations listed in Table 2). All these algorithms use the triangle thresholding method. Moreover, T4 and T8 frequently failed to segment images; therefore, we recommend against using them for automatic fibrin clot analysis. Algorithms M1, M2, M3, S2, S5, and S6 (group 3) returned significantly above average diameter values (above one standard deviation); they all use Statistical Region Merging with a low Q value. The rest of the algorithms S1, S3, S7, M4–M7, T1–T3, and T5–T8 (group 2) return about average values (within one standard deviation of the mean of all algorithms for a given set). 

### 3.1. Comparison of Manual and Automated Fiber Diameter Measurements Using Three Large Data Sets

We performed diameter measurements on three large data sets of 120 SEM images (set 1, 15 participants, 8.28 nm pixel size), 69 SEM images (set 2, 23 participants, 4.9 nm pixel size), and 150 SEM images (set 3, 30 participants, 24.3 nm pixel size). We then compared the manually determined diameter values to the histogram mean and arithmetic mean of diameter values measured by the 24 DiameterJ algorithms. It should be noted that for all three data sets, the histogram mean was on average 10% smaller than the arithmetic mean (this implies a right-skewed diameter distribution, see discussion). We also calculated the correlations between the manually determined diameter measurements and the automated diameter measurements. Complete results are summarized in Supplementary Tables S2–S7, and key results are described below. 

#### 3.1.1. Set 1 Diameter Measurements

The 120 SEM images in set 1 were taken at 10,000× magnification and had a pixel size of 8.28 nm and about 16 pixels per fiber of average thickness. There was excellent agreement (≤5.1% difference) between the diameter values returned by several DiameterJ algorithms (arithmetic mean and histogram mean) and the average manual diameter (133 nm), Table 3 and Table 4. Namely, these algorithms were M4, M5, M6, M7, S3, T1, T2, T3, T5, T6 (arithmetic mean), and M1, M2, M3, S1, S2, S3, S5, S6, S7, T5 (histogram mean). The first list (well-matched arithmetic mean) are algorithms that generally return about average diameter values (group 2); the latter list (well-matched histogram mean) are algorithms that return about average (group 2) and above average values (group 3). In addition, the correlation between the manual diameter values and the diameter values returned by several of the algorithms was very high (*r* > 0.84), Table 3. Particularly, DiameterJ algorithm M6 outperformed other algorithms in correlating with manual measurements (*r* = 0.87) and rendering diameter values within 1 nm of the manual measurements (less than 1% difference). In summary, for this data set, the diameter values returned by numerous algorithms closely agreed and highly correlated with the manual value. The best algorithms are the ones that return about average diameter values in the ranking of algorithms (Table 2). 

#### 3.1.2. Set 2 Diameter Measurements

The 69 SEM images in set 2 were taken at 20,000× magnification and had a pixel size of 4.9 nm and about 25 pixels per fiber of average thickness. In this data set, most algorithms returned diameter values that were lower than the manual diameter value (127 nm); this is true for all 24 histogram means and 20 arithmetic means. Accordingly, algorithms that return above average diameter values (group 3) come closest to the manually determined diameter values: S1, S2, S5, S6, S7, M1, M2, M3 (arithmetic mean) were within 5% (Table 3), and M1, M2 (histogram mean) were within 10% of the manual diameter value. Of these algorithms, all showed better than *r* = 0.71 correlation (arithmetic mean) (Table 3) and better than *r* = 0.67 (histogram mean). 

#### 3.1.3. Set 3 Diameter Measurements

The 150 SEM images in set 3 were taken at 12,000× magnification and had a pixel size of 24.3 nm and about 8 pixels per fiber of average thickness. In this data set, all algorithms returned diameter values (histogram mean and arithmetic mean) that were larger than the manual diameter (191 nm). Here, algorithms that tend to return below average diameter values (group 1) performed best: S4 (arithmetic mean), and M4, M7, M8, S4, S8, T4, and T7 (histogram mean) were within 15% of the manually determined value. Of these, only M7 and T7 (histogram mean) correlated better than *r* = 0.73; though other algorithms show somewhat higher correlations (Table 3 and Table 4). 

The ten DiameterJ algorithms that best correlated with the manually measured diameter values and that were within, 10% (set 1), 15% (set 2), and 35% (set 3) of the manually determined values are given in Table 3 (arithmetic mean) and Table 4 (histogram mean). 

There is surprisingly little overlap in the algorithms that perform best for the three different data sets. The main difference in the three sets is the pixel size of the images, which is determined by the SEM settings (magnification, resolution) and the pixel density of the saved image. The pixel size appears to influence the segmentation and, thus, the diameter output of the algorithms. The smaller the pixel size, the smaller the average diameter that is returned by the algorithms, as compared to the manual measurement. This becomes clear when comparing the averaged automated diameter outputs (averaged over all algorithms) with the manual measurement. In set 2 (smallest pixel size at 4.9 nm), the average of the automatically determined diameter values is 24.9% smaller (histogram mean) and 14.5% smaller (arithmetic mean) than the manual value. In set 1 (8.28 nm pixel size), the average diameter is 7.3% smaller (histogram mean) and 1% larger (arithmetic mean) than the manually determined diameter. In set 3 (24.3 nm pixel size), the average is 17.3% larger (histogram mean) and 30.5% larger (arithmetic mean) than the manual value. A plot of this trend—difference in diameter values between automated measurements and manual measurements versus pixel size—is shown in Supplementary Figure S4. 

To further investigate and validate this trend, we analyzed a subset of 50 images from data set 2. These images were taken of exactly the same clot area as the original data set 2, but with half the magnification (10,000× magnification), and, thus, twice the pixel size (9.9 nm). Confirming the trend, the average of the algorithm-determined diameter values increased in this subset, so that the new average of the automatically determined diameter values is now 6.1% smaller (histogram mean) and 8.7% larger (arithmetic mean) than the manual value (manual value obtained for 20,000× images). These values are close to the values for data set 1, for which the pixel size is 8.28 nm. Moreover, the best-performing algorithms, for which the automatically determined diameter values are within 5% of the manual value, now also changed to a list that strongly overlaps with set 1. These are M5, M6, M7, S8, T1, T2, T3, T6, T7, T8 (arithmetic mean), and M1, M3, M4, S1, S2, S3, S5, S6, S7, T5 (histogram mean). Possible reasons for the pixel size-dependence of the diameter values are discussed in the discussion section.

### 3.2. Detecting Differences between Patient and Healthy Control Plasma Clot Samples 

Having identified the algorithms whose diameter outputs best correlated with manually measured diameter values, we evaluated if these algorithms can be used to distinguish differences in fiber diameters between healthy control and patient samples. We used samples from two different studies, each including a healthy control group.

#### 3.2.1. Postpartum Hemorrhage, Sample Set 1

**Figure 2:**
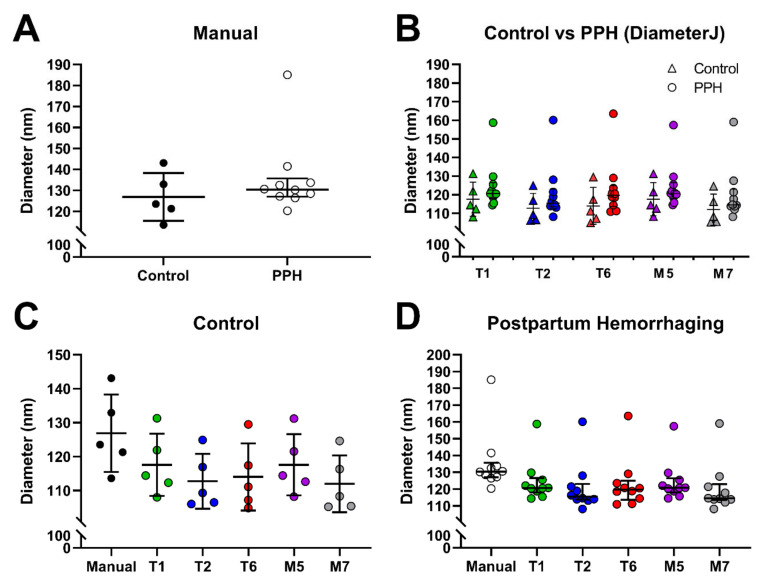
DiameterJ measurements (arithmetic mean, algorithms T1, T2, T6, M5, M7) and manual measurements for five healthy control samples and ten postpartum hemorrhage (PPH) patient samples. (**A**) Manual measurements, and (**B**) automated DiameterJ measurements of healthy control and PPH patient samples. (**C**,**D**) Comparison between manual measurements and automated DiameterJ measurements of healthy control samples (**C**), and PPH patients (**D**). No statistically significant difference in fiber diameter between healthy control samples and PPH samples was observed.

We first compared SEM images from women with postpartum hemorrhage (PPH) and healthy controls. This sample set consisted of ten women with severe postpartum hemorrhage (defined as blood loss of ≥2000 mL within 24 h after delivery) [42] and five controls with uncomplicated deliveries. Eight SEM images from each subject were analyzed for a total of 120 images. The images were analyzed with algorithms M5, M7, T1, T2, and T6, which showed strong, positive correlations (*r* > 0.84) with the manual diameter values and they returned diameter values within 6 nm of the manual measurements (less than 5% difference), Figure 2. In this study, there was no statistically significant difference in fiber diameter between clots from women with postpartum hemorrhage when compared to healthy controls using either the manual measurement or the automated algorithms (arithmetic mean). The manually determined average diameter value was 127 nm for healthy controls versus 136 nm for the hemorrhaging patients (arithmetic mean). This result was also reproduced in the automated measurements, both in terms of statistical significance (no difference) and trend (healthy controls tend to have thinner fibers), Figure 2. 

#### 3.2.2. Systemic Lupus Erythematosus (SLE), Sample Set 4

**Figure 3:**
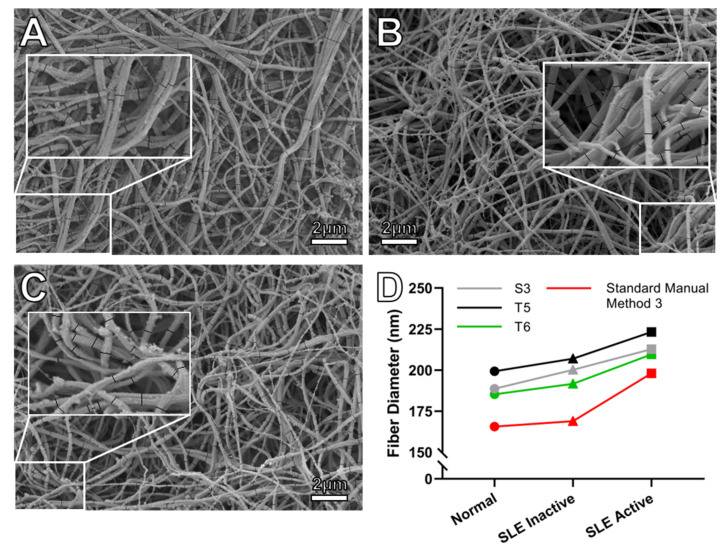
SEM analysis of plasma clots from Systematic Lupus Erythematosus (SLE) patients. SEM images of plasma clots from active SLE patients (**A**), inactive SLE patients (**B**), and healthy controls (**C**). (**D**) Comparison between manual and DiameterJ algorithms measurements of fibrin fiber diameters in active and inactive SLE, and healthy controls. In both manual and the three automated measurements, active SLE samples had a larger diameter than the inactive SLE samples and the healthy controls.

Next, we investigated SEM images from active and inactive systemic lupus erythematosus (SLE) patients [24]. In this study, fibrin fibers in plasma clots from patients with active SLE had a larger diameter, compared to fibers in clots from patients with inactive SLE and healthy controls, when measured manually (Figure 3). We compared SEM images of clots formed from the plasma of healthy individuals (3 SEM images), inactive SLE (5 SEM images), and active SLE (3 SEM images) patients. Images were taken at 5000× magnification, with a pixel size of 22.2 nm (about 8 pixels per average fiber). These imaging parameters are similar to those in set 3, in terms of contrast and pixel size (set 3, pixel size, 24.3 nm). In set 3, the histogram mean correlated better with manual measurements, and the three algorithms T5, T6, and S3 correlated well (*r* > 0.7) with the manually measured diameters and they returned diameter values within 27% of manual measurements. For the manually measured diameters, inactive SLE and healthy controls did not show a significant difference in fiber diameter, though the diameter is trending higher in the active SLE group. The same observed differences among inactive SLE, active SLE, and controls were closely reproduced by algorithms T5, T6, and S3. It should be noted that comparisons were made using the number of images, not the number of patients, as the sample size is relatively small, not allowing the use of statistical analysis. 

The correlations between manual measurements and the diameter values determined by these three algorithms (T5, T6, and S3) are tabulated in Table 5. Again, on average, the arithmetic mean is larger than histogram mean (they both overestimate diameter values) and histogram mean correlates better with manually measured diameter values than the arithmetic mean. 

### 3.3. Relation between Fibrin Fiber Diameter and Biophysical Clot Properties 

To evaluate the performance of automated fibrin clot measurements further, we investigated correlations between biophysical clot properties and the manual and automated diameter measurements (Table 6, top section). Data for biophysical clot properties were only available for data set 3 (N = 30 individuals). The manually determined diameter modestly correlates to fibrinogen concentration (r = 0.44, *p* < 0.01) and to maximum absorbance (r = 0.46, *p* < 0.01). It does not correlate with permeability, storage modulus G’, loss modulus G’’, or tangent δ (G’’/G’). Similar correlations were found between the automated diameter measurements and these biophysical properties. There was a modest correlation between the automated fiber diameter and fibrinogen concentration and maximum absorbance. The diameter values measured by T4 and M8 algorithms show the highest correlations with fibrinogen concentration (r = 0.58 and r = 0.51, respectively). There was no correlation between the automated fiber diameter measurements and the other biophysical properties (permeability, storage modulus G’, loss modulus G’’ or G’’/G’). Table 6 lists correlations for selected algorithms, Supplementary Table S8 provides the full list of algorithms. Overall, manual and automated diameter measurements showed matching moderately positive or null correlations to biophysical clot properties. This is an indirect indication that the automated diameter determinations can perform reliably. 

### 3.4. Relation between Fibrin Network Porosity and Biophysical Clot Properties 

DiameterJ can also be used to systematically determine the mean pore area and the percent porosity of fibrin meshes. However, the mean pore area and porosity of fibrin meshes are difficult to determine because they depend on the depth of field of the image. Since the pore area is determined from a two-dimensional SEM image, the pore area becomes smaller as more image depth (and, thus, more fibers) is included in the analysis. For an accurate analysis, only the top layer of the clot should be included. The proper depth of field is difficult to determine in manual and in automated porosity measures. According to DiameterJ guidelines, T5 and T6 segmentation yield the most visually representative binary version to the original SEM images. In agreement with this, the pore area and percent porosity as determined by T5 (*r* = 0.41 and *r* = 0.42) and T6 (*r* = 0.37 and *r* = 0.41) also demonstrated the highest correlation with permeability, *K_s_*, of all 24 segmentation algorithms (Supplementary Table S9).

Table 6 (bottom section) presents the correlations between porosity measurements—experimentally determined permeability and DiameterJ-determined mean pore area and percent porosity and biophysical measures of the clot-fibrinogen concentration, maximum absorbance, permeability, storage modulus, loss modulus, and the ratio of the moduli (tangent δ, G’’/G’). Both the mean pore area and the percentage porosity obtained with T5 and T6 correlated moderately with permeability (*K_s_*). While the calculated mean pore area did not correlate with fibrinogen concentration, the percentage porosity and permeability, *K_s_*, had a similar association with fibrinogen concentration (T5: *r* = −0.50; *p* = 0.001 and *r* = −0.54; *p* = 0.003, respectively). Neither mean pore area nor percentage porosity, however, demonstrated any association with maximum absorbance or clot mechanical properties, while permeability (*K_s_*) demonstrated significant negative correlations with both.

## 4. Discussion

A mesh of fibrin fibers with diameters ranging from about 100 nm to 200 nm is the major structural component and mechanical scaffold of a blood clot. Microscopic structural parameters of this fibrin mesh, such as fiber diameter, length, fiber and branch point density and clot porosity, affect overall macroscopic clot properties, such as clot viscoelasticity and clot lysis [43,44,45,46]. Microscopic clot properties have also been associated with thrombotic disease [11,21,47,48] and bleeding disorders [16,18,19,20,22,23]. This relationship between microscopic and macroscopic clot properties and diseases is complex and an active area of investigation [21,46,49,50,51]. An unbiased, reliable method to determine microscopic clot parameters would accelerate and facilitate these investigations. SEM is a powerful, widely used, and direct technique to analyze the microscopic structural features of fibrin networks. Although the preparation of clots for SEM, involving fixation, dehydration, drying, and sputter coating, may induce slight changes in clot structure, measurements of fiber diameter and porosity are reproducible and comparisons between clots of patients and controls are highly informative. Moreover, accurate measurements of these properties by light microscopy are not possible because the resolution is not adequate. Currently, the clot structure in SEM images is mostly analyzed manually, which is tedious and may introduce user bias. An automated analysis program for SEM images would reduce tedium and user bias in clot microstructure analysis. It may also be useful in developing a standardized analysis protocol of fibrin clot microstructure. A further advantage of automated measurements is that large data sets could be processed quickly and easily. The aim of our study was to evaluate the image analysis program DiameterJ for fibrin network analysis, in particular, fiber diameter and clot porosity measurements. 

Across the included sets of images, we found that the algorithms can be ranked by diameter output (Table 2), with some algorithms (S4, S8, T4, M8) producing smaller diameter values, some (S1, S3, S7, M4–M7, T1–T3, T5–T8) about average diameter values and some (M1–M3, S2, S5, S6) larger diameter values. We also found that the pixel size of the SEM images critically influences segmentation. Smaller pixels result in smaller diameter values, even for the same data set (e.g., data set 2 with smaller and larger pixels). These two factors (ranking of algorithms and pixel size) are critical for selecting the proper algorithms for analyzing SEM images of fibrin clots. These factors may also explain why there is little overlap of best algorithms across data sets, as different algorithms perform best for data sets with different pixel sizes. 

### 4.1. Optimal Image Parameters and Algorithms

Our results indicate that the optimal combination of image parameters and algorithms is as follows: 1) images with a pixel size of about 8 to 10 nm (about 13 to 16 pixels per average fiber diameter) and 2) algorithms that output about average diameters. This was seen in data set 1 (pixel size 8.28 nm) and the data 2 subset (pixel size 9.9 nm), as several algorithms performed very well. These algorithms were M5–M7, T1–T3, T6 (arithmetic mean), and M1–M3, S1–S3, S5–S7, T5 (histogram mean (Gaussian peak value)). This combination provided accurate diameter values (≤5% within manual values) and high correlations with the manual diameter value (*r* ≥ 0.8 for data set 1). Based on these observations, we recommend this combination (and the corresponding SEM settings) as optimal for the automated analysis of fibrin fiber diameter. Based on set 1, the best among these are M6, M5, and T3, having very close diameter values to the manual measurements (within 1 nm, or ~1%), and also showing strong correlations with the standard manual method (*r_M6_* = 0.87, *r_M5_* = 0.84, *r_T3_* = 0.84). The Huang thresholding method is used in algorithm M6 while M5 and T3 utilize the Minimum Error technique for segmentation (Supplementary Table S1). 

When images with a smaller pixel size are analyzed (e.g., set 2, pixel size 4.9 nm, 25 pixels per average fiber), DiameterJ algorithms tend to return smaller diameter values when compared to manual measurements. For images with larger pixel sizes (e.g., set 3 and the SLE set (set 4), pixel size 24.3 nm and 22.2 nm, 8 pixels per average fiber), DiameterJ algorithms tend to return larger diameter values. Therefore, algorithms that return above average diameter values (e.g., S2, M2, and M1) work best for sets with small pixels (data set 2), and algorithms that return below average values (M4, S4, S8, T4) work best for sets with larger pixels (set 3 and SLE set 4). However, in this latter case, segmentation is starting to be compromised (i.e., there are large gaps in segmentation), which affects correlation. Thus, while algorithms T5 and T6 exaggerated the diameter measurements, they correlated best with manual measurements in set 3 (> 0.81 for the histogram mean and > 0.77 for the arithmetic mean). From these observations, it appears that DiameterJ algorithms start to fail at pixel sizes ≥ 24 nm (8 pixels per average fiber). 

The histogram mean (peak of Gaussian fitted to diameter distribution) was about 10% smaller than the arithmetic mean for all sets. Therefore, the histogram mean was closer to the manual diameter measurement for sets with large pixels, and the arithmetic mean was more accurate for images with smaller pixels. The observation that the histogram mean (Gaussian peak) was 10% smaller also implies that the diameter distribution is not Gaussian and has a tail toward large diameter values (right-skewed). 

In a recent study, DiameterJ was used to determine fiber diameter in blood clots formed in different platelet preparations [52]. Images were taken with a pixel size of 20 nm (5000× magnification), similar to the 24.3 nm and 22.2 nm pixel size of sets 3 and 4. The average diameter in this study was 213 nm over 160 clots. While the specifically used algorithm was not reported in this study, the obtained diameter value is consistent with the generally larger than 200 nm diameter values obtained for sets 3 and 4. This suggests again that larger pixel sizes results in larger diameter values. 

### 4.2. Possible Reasons for Pixel Dependence of Diameter Measurement

Inspecting the segmented images of set 2 at the smaller and larger pixel sizes suggests two underlying reasons for the observed pixel size dependence of the diameter measurements. 1) When two fibers are laterally bundled or very close to each other, the segmentation algorithms tend to combine these two fibers into one larger fiber, thus increasing the measured fiber diameter (Supplementary Figure S6A,B). This effect increases with increasing pixel size. 2) When processing images with smaller pixel sizes, segmentation algorithms tend to include fibers that are deeper in the clot but render them as thin, incomplete fibers. This phenomenon is more prevalent in images with the smaller pixel size (Supplementary Figure S6C,D), thus decreasing the measured diameter for these images. 

Ultimately, in both mechanisms, the resolution relative to the diameter of the fiber (number of pixels per fiber diameter) is the key factor that contributes to the accuracy of algorithms in determining diameter values.

In manual measurements, ImageJ smooths the transition between pixels, and the measured diameter values are no longer integer multiples of the pixel size. That is, the user can choose the starting and ending points of a diameter measurement to be inside a pixel (not necessarily at the edge of a pixel). We did not investigate how pixel size affects manual measurements. Such an investigation would involve manually measuring fiber diameter in images of the same sample that were taken at different resolutions. After binarization (segmentation), DiameterJ always begins and ends the diameter measurement at the edge, between a black and white pixel. Thus, the diameter is always an integer multiple of the corresponding pixel size. For instance, for the 5000× magnification (Figure 3, pixel size 22.2 nm), the diameter is an integer multiple of 44.4 nm. (DiameterJ measures the radius; diameter is twice the radius). Nevertheless, the mean diameter can be determined much more accurately than the pixel size, when many measurements are taken, as was the case in our study. The precision of a measurement is given by $\sigma /\sqrt{N}$, where σ is the standard deviation and *N* is the number of measurements; in our case σ ~ 25 nm and *N* ~ 625, yielding a precision for the mean diameter of about 1 nm. 

### 4.3. Detecting Statistically Significant Differences in Patient Samples

A key utility test for automated diameter measurement programs is whether they can reliably distinguish between control and (pro)thrombotic or hemorrhagic patient samples that have different fiber diameters. Our data suggest that when using algorithms that are appropriate for the image pixel size, differences can be detected. In the two available patient samples (postpartum hemorrhage (PPH) and systemic lupus erythematosus (SLE)), there was no statistical difference in the manually determined diameter between patients and the control sample. However, a clear trend was observed between patients and controls in both sample sets. DiameterJ analysis reproduced both tendencies correctly—it detected no statistical difference and closely matched the trend. Algorithms M6, M5, and T3 (top performers for set 1) closely matched the manual measurements in PPH samples (Figure 2). 

For the lupus samples (large pixel size), for which there is a strong trend for SLE samples to have larger fibers compared to the healthy controls, algorithms T6, T5, and S3 (the best algorithms for lower resolution SEM images with larger pixel size), successfully reproduced the manual measurements (Figure 3). The sample size was, however, small in the SLE set, and it is likely that with a larger sample size a statistical significance would have been detected. 

### 4.4. Correlation of Diameter Measurements with Biophysical Clot Properties

The structure of a fibrin clot must also correlate with its biophysical properties. In the first set of these experiments, we investigated if the diameter correlates with fibrinogen concentration, maximum optical absorbance (turbidity), clot permeability (*K_s_*), storage modulus (G’), loss modulus (G’’), and the loss tangent or tangent δ (G’’/G’) (Supplementary Table S8) across 30 samples (set 3). The manually measured diameter and diameters determined by T6, T5, S7, and M7 all correlated moderately with maximum absorbance. A correlation would be expected since according to light scattering theory, maximum absorbance increases with increasing diameter [53,54,55,56,57]. Fiber diameter did not correlate with clot permeability, G’, G’’, or G’’/G’ for any of the other measurements, likely because these properties are related in a complex manner to other aspects of fibrin network structure, such as branching, in addition to diameter. Agreement in correlations and non-correlations between diameter measurements performed manually and automatically and clot biophysical properties increase confidence that the automated diameter measurements perform accurately. 

### 4.5. Correlation between Fibrin Network Porosity and Biophysical Clot Properties

The porosity of a clot may relate to disease; reduced porosity is associated with a prothrombotic phenotype [3,58], whereas higher porosity is associated with an antithrombotic phenotype [16,21]. DiameterJ has functionality that can determine quantities that reflect the fibrin network porosity. However, this comes with the caveat that it is challenging to determine the porosity of a three-dimensional structure from two-dimensional images. This is because in two-dimensional images the open area between fibers decreases, as more fibers that are deeper in the clot are included in the analysis, thereby reducing the pore size. Thus, only fibers on the surface of the imaged clot sample (not deep into the clot) should be included in this analysis; however, determining which fibers should be included is challenging for automated algorithms (and to a lesser extent for manual image analysis, as well). Visual inspection suggested that the T5 and T6 algorithms best match the clot structure (set 3), and these algorithms, thus, performed best in the fibrin network porosity analysis. 

The DiameterJ-determined mean pore area and percent porosity only weakly correlated with the experimentally determined clot permeability (*K_s_*) which is the method of choice to analyze pore size in fibrin clots. The percent porosity, as determined by the T5 and T6 algorithms, however, demonstrated a similar association with fibrinogen concentration as *K_s_*, and it may, therefore, be a credible measure of clot porosity. The mean pore area as determined by the T5 and T6 algorithms did not correlate as well with the other measurements as *K_s_* did, and only moderately correlated with *K_s_* itself. A confounding factor in this analysis is the fact that the mean pore area, as calculated from the SEM images, is obtained from a dehydrated sample, whereas *K_s_* is obtained from, and compared to, biophysical clot properties that are obtained from fully hydrated clots. We advise caution when using automated fibrin network porosity measurements. A key challenge for porosity analysis is determining the proper analysis depth in the two-dimensional SEM images. 

### 4.6. Limitation Due to Particles in Sample

We also applied the DiameterJ algorithms to a sample of 140 SEM images from healthy controls [31]. As shown in Supplementary Figure S5, aggregates of particles attached to fibrin fibers, which resulted in poor segmentation and overestimation of fiber diameter measurements (the origin of the particles in these images in not known). Thus, having aggregates or particles in the fibrin clot images will compromise analysis. 

## 5. Conclusions

The overall aim of our study was to test which algorithms of the ImageJ plug-in, DiameterJ, can accurately and automatically analyze fiber diameters and porosity of fibrin clots in SEM images. We conclude that SEM images with a pixel size of 8 to 10 nm (13 to 16 pixels per average fiber diameter) are optimal and several algorithms (M6, M5, T3 are best) return highly accurate diameter values (arithmetic mean) that also demonstrate strong correlations with manually measured diameter values. Properly selected DiameterJ algorithms correctly reproduced diameter trends in control samples compared to samples from patients with hemostatic disorders. When the pixel size is larger or smaller than the optimal 8 to 10 nm, DiameterJ algorithms tend to overestimate and underestimate diameter values, respectively. Furthermore, manual and automated diameter measurements showed the same correlations (or absence of correlations) to biophysical clot properties (maximum optical absorbance, liquid permeability, and the parameters of viscoelasticity). Due to difficulties in determining image depth of field, it is challenging to determine network porosity measures via DiameterJ. Thus, the correlation between DiameterJ determined fibrin clot porosity measures (pore size and percent porosity) and permeability was only moderate. 

## Figures and Tables

**Table 1:** Demographic data of study population. Data are listed as median (range), or %, as appropriate. A bleeding score was calculated using the International Society on Thrombosis and Haemostasis Bleeding Assessment Tool (ISTH-BAT), with a cut-off score of ≥ 6 for women.

Characteristics	Women with Severe PPH (*n* = 10)	Women without PPH (*n* = 5)	*p*
Age, year	31 (25–33)	43 (36–44)	0.01
N of pregnancies	3 (2–4)	2 (1–3)	0.61
N of miscarriages	1 (0–2)	0 (0–1)	0.24
Blood loss, mL	3000 (2000–7000)	<500	n/a
Blood type O, %	33%	50%	0.57
Abnormal bleeding score, %	60%	0%	0.03

**Table 2:** Average deviation from the arithmetic mean for each algorithm, across sets 1, 2, 3.

Algorithms	S4	M8	T4	S8	T8	T7	M7	T2
Average deviations from arithmetic mean (%)	−13.03	−12.33	−12.29	−8.46	−6.16	−6.09	−5.91	−5.72
**Algorithms**	**M4**	**T6**	**T3**	**M6**	**M5**	**T1**	**T5**	**S3**
Average deviations from arithmetic mean (%)	−4.24	−2.72	−1.87	−1.76	−1.37	−1.11	4.77	5.09
**Algorithms**	**S1**	**S7**	**M3**	**S2**	**S5**	**S6**	**M1**	**M2**
Average deviations from arithmetic mean (%)	7.37	7.61	8.47	8.76	8.93	11.06	11.63	13.80

**Table 3:** Correlations and arithmetic mean values for the 10 DiameterJ algorithms that correlated best with the manual measurements (arithmetic mean). Diameter values are within 10% (set 1), 15% (set 2), and 35% (set 3) of manual measurements, respectively. * *p* < 0.0001 for all.

Type of Measurement	Manual	M6	M7	M3	M5	T2	T6	T3	T4	T5	S7
Arithmetic Mean for set 1; N = 120 SEM images (nm)	133	132	127	140	133	128	130	132	120	138	142
Correlation *r* * with Manual Method	-	0.87	0.86	0.85	0.85	0.85	0.85	0.85	0.85	0.85	0.85
	**Manual**	**S2**	**M2**	**M1**	**S5**	**S6**	**S1**	**M3**	**M4**	**S7**	**S3**
Arithmetic Mean for set 2; N = 69 SEM images (nm)	127	122	131	128	123	127	121	128	111	124	119
Correlation *r* * with Manual Method		0.77	0.76	0.76	0.75	0.75	0.73	0.72	0.71	0.71	0.66
	**Manual**	**T6**	**T1**	**M5**	**T2**	**M7**	**S3**	**M3**	**T7**	**S7**	**T8**
Arithmetic Mean for set 3; N = 150 SEM images (nm)	191	249	253	253	235	235	250	254	245	253	244
Correlation *r* * with Manual Method	-	0.77	0.74	0.74	0.73	0.73	0.72	0.71	0.70	0.70	0.68

**Table 4:** Correlations and histogram mean values for the 10 DiameterJ algorithms that correlated best with the manual measurements (arithmetic mean). Diameter values are within 10% (set 1), 15% (set 2), and 35% (set 3) of manual measurements, respectively. * *p* < 0.0001 for all.

Type of Measurement	Manual	T1	S5	M6	S3	M1	S7	M3	T5	S1	T6
Histogram Mean for set 1; N = 120 SEM images (nm)	133	122	135	121	129	135	131	130	126	135	120
Correlation *r* * with Manual Method	-	0.85	0.85	0.85	0.85	0.85	0.84	0.84	0.82	0.82	0.81
	**Manual**	**S1**	**M2**	**S5**	**M1**	**S2**	**S6**	**M3**	**S7**	**S3**	**M4**
Histogram Mean for set 2; N = 69 SEM images (nm)	127	108	116	110	115	109	113	113	111	106	100
Correlation *r* * with Manual Method		0.69	0.69	0.68	0.67	0.67	0.66	0.66	0.62	0.61	0.60
	**Manual**	**T6**	**S3**	**M5**	**M3**	**T1**	**T2**	**T7**	**S7**	**T8**	**M7**
Histogram Mean for set 3; N = 150 SEM images (nm)	191	225	222	229	227	228	214	223	221	221	214
Correlation *r* * with Manual Method	-	0.82	0.80	0.79	0.79	0.79	0.79	0.78	0.79	0.74	0.73

**Table 5:** Correlations between manual diameter measurements (arithmetic mean) and the diameter values determined by DiameterJ algorithms T6, T5, and S3 for 11 SEM images of clots made from normal, inactive, and active SLE patients.

Type of Measurement	Manual	T6	T5	S3
Arithmetic Mean; *N* = 11	178	191	234	230
Correlation *r*(*p*) between the arithmetic mean and manual measurement	-	0.73 (0.01)	0.68 (0.02)	0.83 (0.001)
Histogram Mean; *N* = 11	178	192	206	197
Correlation *r*(*p*) between the histogram mean and manual measurement	-	0.80 (0.003)	0.72 (0.01)	0.78 (0.004)

**Table 6:** Correlations between diameter (manual and automated) and biophysical clot properties (top). Correlations between porosity measures (permeability (K_s_), automated pore size and automated porosity) and biophysical clot properties (N = 30 participants, 150 images).

Variable	Fibrinogen Concentration *r* (*p*)	Maximum Absorbance *r* (*p*)	Permeability (*K_s_*) *r* (*p*)	Storage Modulus (G’) *r* (*p*)	Loss Modulus (G”) *r* (*p*)	Tan Delta (G’/G”) *r* (*p*)
**Diameter**						
Manual	0.44 (0.01)	0.46 (0.01)	−0.04 (0.9)	0.27 (0.2)	0.29 (0.1)	−0.06 (0.7)
M8	0.51 (0.01)	0.57 (0.006)	−0.16 (0.47)	0.30 (0.18)	0.29 (0.19)	−0.13 (0.57)
T4	0.58 (0.003)	0.58 (0.003)	−0.12 (0.58)	0.23 (0.27)	0.21 (0.32)	−0.12 (0.59)
T6	0.33 (0.07)	0.43 (0.02)	0.03 (0.87)	0.28 (0.14)	0.24 (0.20)	−0.06 (0.73)
T5	0.32 (0.08)	0.42 (0.02)	0.071 (0.70)	0.34 (0.07)	0.28 (0.13)	−0.20 (0.28)
S6	0.25 (0.18)	0.37 (0.05)	0.15 (0.43)	0.34 (0.07)	0.30 (0.10)	−0.18 (0.35)
M6	0.26 (0.17)	0.35 (0.06)	0.12 (0.52)	0.27 (0.15)	0.20 (0.27)	−0.16 (0.40)
**Porosity**						
Permeability *K_s_* (cm^2^ × 10^−9^)	−0.54 (0.003)	−0.67 (<0.0001)	-	−0.48 (0.007)	−0.51 (0.004)	−0.18 (0.4)
T6 Mean pore area (µm^2^)	−0.14 (0.43)	0.08 (0.67)	0.37(0.03)	0.22 (0.24)	0.19 (0.32)	−0.15 (0.17)
T5 Mean pore area (µm^2^)	−0.13 (0.49)	0.08 (0.67)	0.41 (0.02)	0.23 (0.23)	0.22 (0.24)	−0.19 (0.32)
S6 Mean pore area (µm^2^)	−0.11 (0.55)	0.10 (0.61)	0.25 (0.18)	0.21 (0.25)	0.21 (0.27)	−0.00 (0.10)
M6 Mean pore area (µm^2^)	−0.03 (0.88)	0.24 (0.20)	0.10 (0.61)	0.21 (0.28)	0.23 (0.23)	0.06 (0.77)
T6 Porosity (%)	−0.42 (0.02)	−0.25 (0.18)	0.41 (0.02)	0.07 (0.71)	0.08 (0.67)	−0.20 (0.27)
T5 Porosity (%)	−0.50 (0.004)	−0.23 (0.20)	0.42 (0.20)	−0.05 (0.78)	−0.01 (0.96)	−0.05 (0.79)
S6 Porosity (%)	−0.26 (0.17)	−0.28 (0.13)	0.06 (0.75)	−0.17 (0.36)	−0.16 (0.41)	0.12 (0.51)
M6 Porosity (%)	−0.05 (0.80)	0.1 (0.62)	−0.03 (0.89)	0.08 (0.68)	0.13 (0.49)	0.09 (0.64)

## Data Availability

Original data underlying this study may be obtained from the authors upon request.

## Supplementary Materials

Supplementary materials available online at https://www.mdpi.com/article/10.3390/biom11101536/s1, Details on the segmentation algorithms, Figure S1: The overlap between the probability density of object and background in a bimodal distribution used for minimum error thresholding, Figure S2: Finding threshold using Triangle thresholding method, Table S1: The general characterizations of 24 segmentation algorithms in DiameterJ, Table S2: Average histogram means determined by 24 algorithms included in DiameterJ and their correlations with standard manual method 1 for 120 SEM images of PPH and control samples (set 1), Table S3: Average arithmetic means determined by 24 algorithms included in DiameterJ and their correlations with standard manual method 1 for 120 SEM images of PPH and control samples (set 1), Table S4: Average histogram means determined by 24 algorithms included in DiameterJ and their correlations with standard manual method 2 for 69 SEM images of acute MI samples (set 2), Table S5: Average arithmetic means determined by 24 algorithms included in DiameterJ and their correlations with standard manual method 2 for 69 SEM images of acute MI samples (set 2), Table S6: Average histogram means determined by 24 algorithms included in DiameterJ and their correlations with standard manual method 3 for 150 SEM images of plasma samples (set 3), Table S7: Average arithmetic means determined by 24 algorithms included in DiameterJ and their correlations with standard manual method 3 for 150 SEM images of plasma samples (set 3), Table S8: The correlation between diameter values determined by standard manual method 3 and automated fiber diameter measurements and biophysical properties of clots of plasma fibers and (*N* = 30 participants, 150 images), Table S9: The correlation between permeability (K_s_) and automated porosity calculations determined by algorithms included in DiameterJ and biophysical properties of clots of plasma fibers and (*N* = 30 participants, 150 images), Figure S3: Representative images from (A) set 1, (B) set 2 and (C) set 3, and the corresponding distributions of gray scale levels, Figure S4: Difference in diameter values between DiameterJ automated measurements (arithmetic mean, average for all algorithms) and manual measurements versus pixel size, Figure S5: Particles (of unknown origin; possibly microvesicles, especially from platelets, since they have αIIbβ3 in their membranes) attached to fibrin fibers results in poor image segmentation by DiameterJ.

## References

[1] Badiei N., Sowedan A., Curtis D., Brown M., Lawrence M., Campbell A., Sabra A., Evans P., Weisel J., Chernysh I. (2015). Effects of unidirectional flow shear stresses on the formation, fractal microstructure and rigidity of incipient whole blood clots and fibrin gels. Clin. Hemorheol. Microcirc..

[2] Baheti S., Tunak M. (2017). Characterization of fiber diameter using image analysis. IOP Conf. Ser. Mater. Sci. Eng..

[3] Kopytek M., Zabczyk M., Natorska J., Siudut J., Malinowski K.P., Ptaszek P., Glajcar A., Goralczyk T., Undas A. (2019). Viscoelastic properties of plasma fibrin clots are similar in patients on rivaroxaban and vitamin K antagonists. J. Physiol. Pharmacol. Off. J. Pol. Physiol. Soc..

[4] Langer B.G., Weisel J.W., Dinauer P., Nagaswami C., Bell W.R. (1988). Deglycosylation of fibrinogen accelerates polymerization and increases lateral aggregation of fibrin fibers. J. Biol. Chem..

[5] Li W., Sigley J., Pieters M., Helms C.C., Nagaswami C., Weisel J.W., Guthold M. (2016). Fibrin Fiber Stiffness Is Strongly Affected by Fiber Diameter, but Not by Fibrinogen Glycation. Biophys. J..

[6] Longstaff C., Thelwell C., Williams S.C., Silva M.M.C.G., Szabó L., Kolev K. (2011). The interplay between tissue plasminogen activator domains and fibrin structures in the regulation of fibrinolysis: Kinetic and microscopic studies. Blood.

[7] Moreno-Arotzena O., Meier J.G., Del Amo C., García-Aznar J.M. (2015). Characterization of Fibrin and Collagen Gels for Engineering Wound Healing Models. Materials.

[8] Nguyen D.M. (2019). Analysis of Fiber Network Architecture in Blood Vessels and Blood Clots. Master’s Thesis.

[9] Weisel J.W., Litvinov R. (2013). Mechanisms of fibrin polymerization and clinical implications. Blood.

[10] Collet J., Woodhead J., Soria J., Soria C., Mirshahi M., Caen J., Weisel J. (1996). Fibrinogen Dusart: Electron microscopy of molecules, fibers and clots, and viscoelastic properties of clots. Biophys. J..

[11] Collet J., Allali Y., Lesty C., Tanguy M., Silvain J., Ankri A., Blanchet B., Dumaine R., Gianetti J., Payot L. (2006). Altered Fibrin Architecture Is Associated with Hypofibrinolysis and Premature Coronary Atherothrombosis. Arter. Thromb. Vasc. Biol..

[12] Dunn E.J., Ariëns R.A.S. (2004). Fibrinogen and Fibrin Clot Structure in Diabetes. Herz.

[13] Campbell R.A., Overmyer K.A., Selzman C.H., Sheridan B.C., Wolberg A.S. (2009). Contributions of extravascular and intravascular cells to fibrin network formation, structure, and stability. Blood.

[14] Fatah K., Silveira A., Tornvall P., Karpe F., Blombäck M., Hamsten A. (1996). Proneness to Formation of Tight and Rigid Fibrin Gel Structures in Men with Myocardial Infarction at a Young Age. Thromb. Haemost..

[15] Machlus K., Cardenas J.C., Church F.C., Wolberg A.S. (2011). Causal relationship between hyperfibrinogenemia, thrombosis, and resistance to thrombolysis in mice. Blood.

[16] Mikovic D., Elezovic I., Zabczyk M., Hutenby K., Antovic J.P., Antovic A. (2014). Improvement of fibrin clot structure after factor VIII injection in haemophilia A patients treated on demand. Thromb. Haemost..

[17] Bridge K., Philippou H., Ariëns R.A.S. (2014). Clot properties and cardiovascular disease. Thromb. Haemost..

[18] Dargaud Y., Prevost C., Lienhart A., Bordet J.C., Negrier C. (2011). Evaluation of the overall haemostatic effect of recombinant factor VIIa by measuring thrombin generation and stability of fibrin clots. Haemophilia.

[19] Hethershaw E., La Corte A.L.C., Duval C., Ali M., Grant P.J., Ariens R., Philippou H. (2013). The effect of blood coagulation factor XIII on fibrin clot structure and fibrinolysis. J. Thromb. Haemost. JTH.

[20] Leong L., Chernysh I.N., Xu Y., Sim D., Nagaswami C., De Lange Z., Kosolapova S., Cuker A., Kauser K., Weisel J.W. (2017). Clot stability as a determinant of effective factor VIII replacement in hemophilia A. Res. Pract. Thromb. Haemost..

[21] Undas A., Ariëns R.A. (2011). Fibrin Clot Structure and Function: A Role in the Pathophysiology of Arterial and Venous Thromboembolic Diseases, Arterioscler. Arter. Thromb. Vasc. Biol..

[22] Wolberg A.S., Allen G.A., Monroe D.M., Hedner U., Roberts H.R., Hoffman M. (2005). High dose factor VIIa improves clot structure and stability in a model of haemophilia B. Br. J. Haematol..

[23] Zucker M., Seligsohn U., Salomon O., Wolberg A.S. (2014). Abnormal plasma clot structure and stability distinguish bleeding risk in patients with severe factor XI deficiency. J. Thromb. Haemost. JTH.

[24] Litvinov R.I., Nabiullina R.M., Zubairova L.D., Shakurova M.A., Andrianova I.A., Weisel J.W. (2019). Lytic Susceptibility, Structure, and Mechanical Properties of Fibrin in Systemic Lupus Erythematosus. Front. Immunol..

[25] Pretorius E., Steyn H., Engelbrecht M., Swanepoel A.C., Oberholzer H.M. (2011). Differences in fibrin fiber diameters in healthy individuals and thromboembolic ischemic stroke patients. Blood Coagul. Fibrinolysis.

[26] Siebenlist K.R., Mosesson M.W., Hernandez I., Bush L.A., Di Cera E., Shainoff J.R., Di Orio J.P., Stojanovic L. (2005). Studies on the basis for the properties of fibrin produced from fibrinogen-containing γ′ chains. Blood.

[27] Hotaling N.A., Bharti K., Kriel H., Simon C. (2015). DiameterJ: A validated open source nanofiber diameter measurement tool. Biomaterials.

[28] Hotaling N., Bharti K., Kriel H., Simon C. (2015). Dataset for the validation and use of DiameterJ an open source nanofiber diameter measurement tool. Data Brief.

[29] Yoon J.G., Song J. (2017). Adopting automated image analysis tool for fibrin network: Can we obtain clot properties for practical application?. Int. J. Lab. Hematol..

[30] Weisel J., Nagaswami C. (1992). Computer modeling of fibrin polymerization kinetics correlated with electron microscope and turbidity observations: Clot structure and assembly are kinetically controlled. Biophys. J..

[31] Siniarski A., Baker S.R., Duval C., Malinowski K.P., Gajos G., Nessler J., Ariëns R.A. (2021). Quantitative analysis of clot density, fibrin fiber radius, and protofibril packing in acute phase myocardial infarction. Thromb. Res..

[32] Thygesen K., Alpert J.S., Jaffe A.S., Simoons M.L., Chaitman B.R., White H.D. (2012). Third Universal Definition of Myocardial Infarction. Circulation.

[33] Teo K., Chow C.K., Vaz M., Rangarajan S., Yusufali A. (2009). The Prospective Urban Rural Epidemiology (PURE) study: Examining the impact of societal influences on chronic noncommunicable diseases in low-, middle-, and high-income countries. Am. Hear. J..

[34] Pieters M., Philippou H., Undas A., De Lange Z., Rijken D., Mutch N. (2018). An international study on the feasibility of a standardized combined plasma clot turbidity and lysis assay: Communication from the SSC of the ISTH. J. Thromb. Haemost..

[35] Lisman T., De Groot P.G., Meijers J.C.M., Rosendaal F.R. (2005). Reduced plasma fibrinolytic potential is a risk factor for venous thrombosis. Blood.

[36] Pieters M., Undas A., Marchi R., DE Maat M.P.M., Weisel J.W., Ariëns R.A.S. (2012). On behalf of the factor xiii and fibrinogen subcommittee of the scientific and standardisation committee of the international society for thrombosis and Haemostasis, an international study on the standardization of fibrin clot permeability measurement: Methodological considerations and implications for healthy control values. J. Thromb. Haemost. JTH.

[37] Schindelin J., Arganda-Carreras I., Frise E., Kaynig V., Longair M., Pietzsch T., Preibisch S., Rueden C., Saalfeld S., Schmid B. (2012). Fiji: An open-source platform for biological-image analysis. Nat. Chem. Biol..

[38] Schneider C., Rasband W.S., Eliceiri K.W. (2012). NIH Image to ImageJ: 25 years of image analysis. Nat. Methods.

[39] Ajjan R., Standeven K., Khanbhai M., Phoenix F., Gersh K., Weisel J., Kearney M., Ariëns R., Grant P. (2009). Effects of Aspirin on Clot Structure and Fibrinolysis Using a Novel In Vitro Cellular System. Arter. Thromb. Vasc. Biol..

[40] Collet J.-P., Moen J.L., Veklich Y.I., Gorkun O.V., Lord S.T., Montalescot G., Weisel J.W. (2005). The αC domains of fibrinogen affect the structure of the fibrin clot, its physical properties, and its susceptibility to fibrinolysis. Blood.

[41] Hugenholtz G.C., Macrae F., Adelmeijer J., Dulfer S., Porte R.J., Lisman T., Ariens R. (2016). Procoagulant changes in fibrin clot structure in patients with cirrhosis are associated with oxidative modifications of fibrinogen. J. Thromb. Haemost. JTH.

[42] Mavrides E., Allard S., Chandraharan E., Collins P., Green L., Hunt B.J., Riris S., Thomson A.J. (2016). Prevention and Management of Postpartum Haemorrhage: Green-top Guideline No. 52. BJOG Int. J. Obstet. Gynaecol..

[43] Collet J.P., Park D., Lesty C., Soria J., Soria C., Montalescot G., Weisel J.W. (2000). Influence of Fibrin Network Conformation and Fibrin Fiber Diameter on Fibrinolysis Speed. Arter. Thromb. Vasc. Biol..

[44] Hudson N.E. (2017). Biophysical Mechanisms Mediating Fibrin Fiber Lysis. BioMed Res. Int..

[45] Weigandt K.M., Pozzo D.C., Porcar L. (2009). Structure of high density fibrin networks probed with neutron scattering and rheology. Soft Matter.

[46] Weisel J.W., Litvinov R.I. (2017). Fibrin Formation, Structure and Properties. Subcell. Biochem..

[47] Baker S.R., Zabczyk M., Macrae F.L., Duval C., Undas A., Ariëns R.A.S. (2019). Recurrent venous thromboembolism patients form clots with lower elastic modulus than those formed by patients with non-recurrent disease. J. Thromb. Haemost..

[48] Litvinov R.I., Weisel J.W. (2016). What Is the Biological and Clinical Relevance of Fibrin?. Semin. Thromb. Hemost..

[49] Litvinov R.I., Weisel J.W. (2016). Fibrin mechanical properties and their structural origins. Matrix Biol..

[50] Wolberg A.S., Campbell R.A. (2008). Thrombin generation, fibrin clot formation and hemostasis. Transfus. Apher. Sci..

[51] Zhmurov A., Brown A.E., Litvinov P.R.I., Dima R.I., Weisel P.J.W., Barsegov P.V. (2011). Molecular Structural Origins of Clot and Thrombus Mechanical Properties. Blood.

[52] Lee S.-J.J., Nguyen D.M., Grewal H.S., Puligundla C., Saha A.K., Nair P.M., Cap A.P., Ramasubramanian A.K. (2019). Image-based analysis and simulation of the effect of platelet storage temperature on clot mechanics under uniaxial strain. Biomech. Model. Mechanobiol..

[53] Carr M.E., Hermans J. (1978). Size and Density of Fibrin Fibers from Turbidity. Macromolecules.

[54] Dassi C., Seyve L., García X., Bigo E., Marlu R., Caton F., Polack B. (2019). Fibrinography: A Multiwavelength Light-Scattering Assay of Fibrin Structure. HemaSphere.

[55] Ferri F., Calegari G.R., Molteni M., Cardinali B., Magatti D., Rocco M. (2015). Size and Density of Fibers in Fibrin and Other Filamentous Networks from Turbidimetry: Beyond a Revisited Carr–Hermans Method, Accounting for Fractality and Porosity. Macromolecules.

[56] Pieters M., Guthold M., Nunes C.M., De Lange Z. (2019). Interpretation and Validation of Maximum Absorbance Data Obtained from Turbidimetry Analysis of Plasma Clots. Thromb. Haemost..

[57] Yeromonahos C., Polack B., Caton F. (2010). Nanostructure of the Fibrin Clot. Biophys. J..

[58] Undas A. (2017). Prothrombotic Fibrin Clot Phenotype in Patients with Deep Vein Thrombosis and Pulmonary Embolism: A New Risk Factor for Recurrence. BioMed Res. Int..

