# Supplementary material for: Automated Fiber Diameter and Porosity Measurements of Plasma Clots in Scanning Electron Microscopy Images
Source: Biomolecules. 2021 Oct 18;11(10):1536. doi: 10.3390/biom11101536 (PMC8533744; doi:10.3390/biom11101536)
Supplement: Supplementary file 1 [file biomolecules-11-01536-s001.zip › biomolecules-1391115-supplementary.pdf]

Supplemental Information

Diameter J algorithms and thresholding techniques

Statistical Region Merging

Let's assume R represents the entire image region. Using the Statistical Region Merging (SRM) segmentation technique, R is partitioned into subregions R_1, $R_{2...}$, R_Q based on their intensity or color. The algorithm starts with a set of random seed points. A statistical test is then applied to the neighboring points and those that have a similar mean intensity or color are merged into the same region. The key parameter in this segmentation algorithm is Q which is a rough estimate of the number of regions in the image [1].

Li Thresholding method

The Li thresholding technique has been introduced to segment the image based on the gray level histogram; it calculates the optimal threshold that minimizes cross-entropy between the segmented image and the original image through an iterative method. Cross entropy is a way to measure the distance between two probability distributions [2,3].

Minimum Error Thresholding

Consider that the pixels of an image have gray levels, g, running [0, …, L] and the population distributions of background and object are normal with distinct means and standard deviations. The histogram is normalized to a probability distribution whose components give the frequency of occurrence of each gray level in the image. As shown in supplement Fig. S1, the probability density function $\mathrm{P(g)}$ consists of components $\mathrm{p(g|i)}$ (conditional probability of g given i occurs) of the mixture. $\mathrm{p(g|i)}$ is normally distributed with a mean μ_i, standard deviation σ_i and a priori probability P_i and can be calculated as follows:

$$\mathrm{P(g)} = \sum_{i=1}^{2} (P_i) . \mathrm{p(g|i)} \qquad (1)$$

where

$$\mathrm{p(g|i)} = \frac{1}{\sqrt{2\pi}\sigma_i} \exp\left(\frac{(g-\mu_i)^2}{2\sigma_i^2}\right) \qquad (2)$$

Minimum error threshold can be obtained by finding Bayes minimum error τ for which gray level g satisfies the following Bayes decision rule

$$P_1 . \mathrm{p(g|1)} \gtrless P_2 . \mathrm{p(g|2)} \begin{cases} g \leq \tau \\ g > \tau \end{cases} \qquad (3)$$

In this method, a criterion function is introduced which indirectly represents the amount of overlap between the probability distribution of background and object [4].

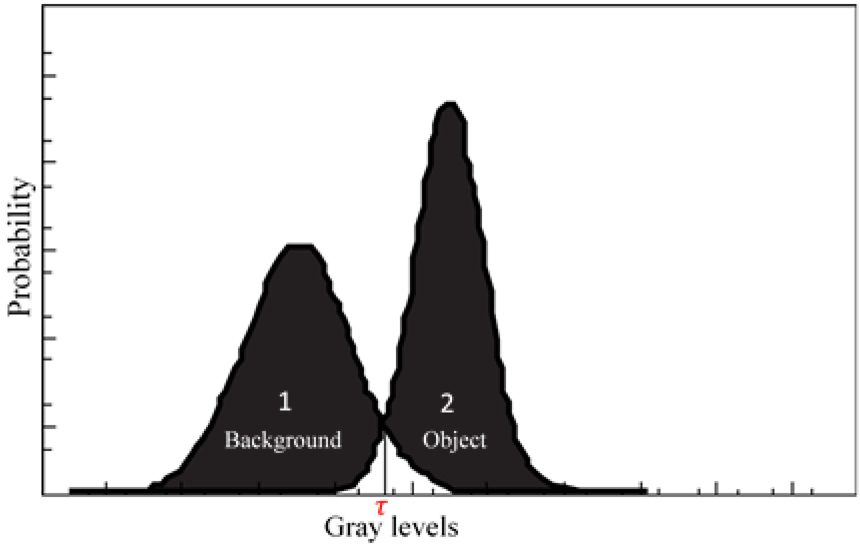

Figure S1. The overlap between the probability density of object and background in a bimodal distribution used for minimum error thresholding [4].

Otsu method

The pixels of an input image are presented in L gray levels [0,…, L]. The histogram of the input image is then normalized into a probability distribution and the components of the histogram are shown by P_i. A threshold K is selected and the pixels are afterward divided into two classes C_1 (the set of pixels with levels [0,…, K]) and C_2 (the set of pixels with levels [K+1,…, L-1]). The between-class variance is defined as

$$\sigma_B{}^2(K) = P_1(K)(\mu_1(K) - \mu_T)^2 - P_2(K)(\mu_2(K) - \mu_T)^2 \qquad (4)$$

Where $P_1(K)$ is the probability of class C_1 occurrence, $P_2(K)$ is the probability of class C_2 occurrence, $\mu_1(K)$ is the mean intensity of class C_1, $\mu_2(K)$ is the mean intensity of class C_2 and μ_T is the global mean intensity of the image. In the Otsu method, the optimal threshold K^* is the value of K which maximizes the between-class variance [5].

Percentile thresholding method

Let's assume the ratio of object area to the total area of the image is known and equal to P%. The percentile method for determining the threshold calculates the threshold as a grey level where the ratio of the number of pixels whose values are higher than the threshold to the total number of pixels in the image is closest to the given percentile (P%) [6].

Triangle thresholding method

The threshold is determined by normalizing the height and the gray levels of the image in the histogram. A line is drawn between the maximum point on the histogram at point M and the lowest point N on the gray level axis as shown in supplement Fig. S2. The normal distance L between the histogram and the line is

calculated for all values between M and N. The gray level at which the normal distance between this line and the histogram is maximal is marked A on the histogram. The threshold value is then determined by adding a fixed offset (typically 0.2) to the value A on the histogram. This value is the new threshold [7].

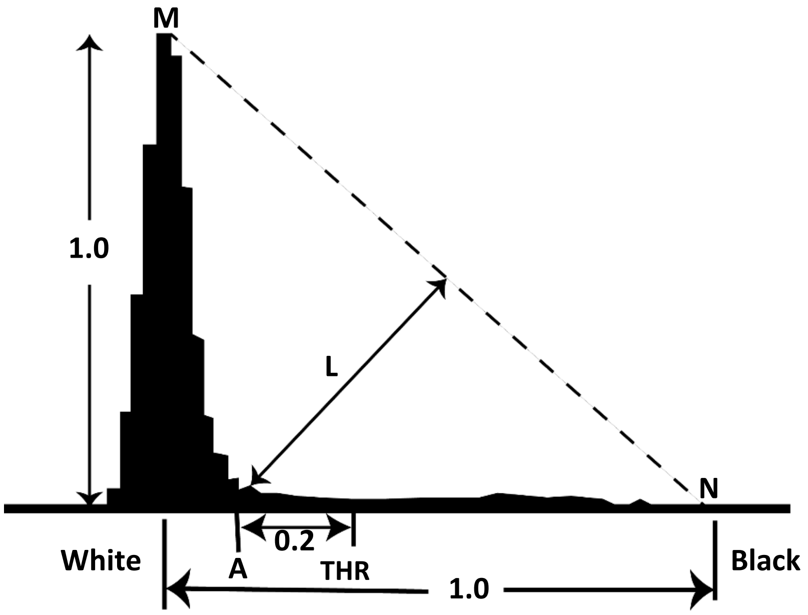

Figure S2. Finding threshold using Triangle thresholding method [7].

Huang's method: Minimizing the measures of fuzziness

Image pixels are divided into background and object by applying the fuzzy set theorem to the image thresholding. The threshold is determined by minimizing the measure of fuzziness in the image. The measure of fuzziness is calculated using entropy and Yagar's method. The threshold can be obtained by minimizing the measure of entropy which uses Shannon entropy to determine the measure of fuzziness in the image. In Yagar's method, the measure of fuzziness, and consequently the threshold, depend on the distance between a fuzzy set and its complement [8].

Characterizations of techniques involved in segmentation

The segmentation algorithms that are implemented in each of the 24 segmentation methods to separate out the objects (fibers) from the background are listed in supplement Table S1. As presented in supplement Table S1, the Traditional category utilizes 8 thresholding techniques (T1-T8) to segment the image into a binary version. The output images are then used to calculate the diameters of fibers.

In the Mixed division, a binary 8-bit image is first extracted from the original image using a region growing segmentation technique (Statistical Region Merging) and then four thresholding techniques (Huang, Minimum Error, Percentile, and Triangle) are applied to this 8-bit image to obtain the final output images

M1-M4. Running a segmentation via the above-mentioned four thresholding techniques (Huang, Minimum Error, Percentile, and Triangle) results in segmented images M5-M8 respectively.

In the statistical region merging group (S1-S8), an integration of a sequence of Statistical Region Merging techniques with a different order of Q and a thresholding technique are used to segment the original SEM image. In S1-S4, the initial merging result is attained using the Statistical region merging technique with Q set to 100. The output is then used as the input for the second merging procedure using Statistical Region Merging with Q of 50 and this will be repeated with smaller Q value 25 followed by the last segmentation with a Q of 12. The resulted segmented images then undergo 4 thresholding methods: Huang, Minimum Error, Percentile, and Triangle.

S5 to S8 segments are obtained using a different sequence of Statistical Region Merging techniques followed by the same four mentioned thresholding methods (Huang, Minimum Error, Percentile, and Triangle). The statistical region merging algorithms with Q = 50 is run first and then the output is used as the input of the second Statistical region merging with Q set to 10. Finally, the four aforementioned thresholding methods are utilized to obtain S5-S8 segments.

Table S1. *The general characterizations of 24 segmentation algorithms in DiameterJ*

Traditional

T1	***T2***	***T3***	***T4***	***T5***	***T6***	***T7***	***T8***
Huang	Percentile	Min Error	Triangle	Li method	Otsu	Maximum entropy	Renyi entropy

Mixed segmentation

M1	***M2***	***M3***	***M4***	***M5***	***M6***	***M7***	***M8***
Statistical Region Merging (Q=25) + Huang	Statistical Region Merging (Q=25) + Min Error	Statistical Region Merging (Q=25) + Percentile	Statistical Region Merging (Q=25) + Triangle	Huang	Min Error	Percentile	Triangle

Statistical Region Merging

S1	***S2***	***S3***	***S4***	***S5***	***S6***	***S7***	***S8***
Statistical Region Merging with 25 gray levels (Q = 100) + Statistical Region Merging with 25 gray levels (Q = 50) + Statistical Region Merging with 25 gray levels (Q = 25) + Statistical Region Merging with 25 gray levels (Q = 12) **+**				Statistical Region Merging with 25 gray levels (Q = 50) + Statistical Region Merging with 25 gray levels (Q = 10) **+**			
Huang	Min Error	Percentile	Triangle	Huang	Min Error	Percentile	Triangle

Table S2. *Average histogram means determined by 24 algorithms included in DiameterJ and their correlations with standard manual method 1 for 120 SEM images of PPH and control samples (set 1).*

	Standard method 1	M1	M2	M3	M4	M5	M6	M7	M8
Histogram Mean for 120 SEM images of hemorrhagic samples (nm)(N=120)	**133**	134	136	130	119	122	121	117	106
Correlation r(p) (Standard method 1)		0.84	0.78	0.83	0.56	0.84	0.85	0.81	0.62
		**S1**	**S2**	**S3**	**S4**	**S5**	**S6**	**S7**	**S8**
Histogram Mean for 120 SEM images of hemorrhagic samples (nm)(N=120)		135	136	129	111	135	136	131	114
Correlation r* (Standard method 1)		0.82	0.78	0.85	0.60	0.85	0.79	0.84	0.36
		**T1**	**T2**	**T3**	**T4**	**T5**	**T6**	**T7**	**T8**
Histogram Mean for 120 SEM images of hemorrhagic samples (nm)(N=120)		122	118	121	109	126	120	112	112
Correlation r* (Standard method 1)		0.85	0.81	0.83	0.79	0.82	0.81	0.60	0.54

**** p<0.0001 for all***

Table S3. *Average arithmetic means determined by 24 algorithms included in DiameterJ and their correlations with standard manual method 1 for 120 SEM images of PPH and control samples (set 1).*

	Standard method 2	M1	M2	M3	M4	M5	M6	M7	M8
Arithmetic Mean for 120 SEM images of hemorrhagic samples (nm)(N=120)	**133**	147	149	140	126	133	132	127	119
Correlation r(p) (Standard method 1)		0.80	0.72	0.85	0.63	0.84	0.87	0.86	0.77
		**S1**	**S2**	**S3**	**S4**	**S5**	**S6**	**S7**	**S8**
Arithmetic Mean for 120 SEM images of hemorrhagic samples (nm)(N=120)		148	150	139	121	148	151	142	123
Correlation r(p) (Standard method 1)		0.75	0.68	0.84	0.50	0.81	0.70	0.85	0.58
		**T1**	**T2**	**T3**	**T4**	**T5**	**T6**	**T7**	**T8**
Arithmetic Mean for 120 SEM images of hemorrhagic samples (nm)(N=120)		133	128	132	120	138	130	122	122
Correlation r(p) (Standard method 1)		0.84	0.85	0.85	0.85	0.82	0.85	0.63	0.68

**** p<0.0001 for all***

Table S4. *Average histogram means determined by 24 algorithms included in DiameterJ and their correlations with Standard manual method 2 for 69 SEM images of acute MI samples (set 2).*

	Standard method 2	M1	M2	M3	M4	M5	M6	M7	M8
Histogram Mean for 69 SEM images of MI samples (nm)(N = 69)	127	115	116	113	100	88	86	81	78
Correlation r(p) (Standard method 2)		0.67(<0.0001)	0.69(<0.0001)	0.66(<0.0001)	0.60(<0.0001)	0.36(0.004)	0.30(0.02)	0.10(0.4)	0.50(0.002)
		**S1**	**S2**	**S3**	**S4**	**S5**	**S6**	**S7**	**S8**
Histogram Mean for 69 SEM images of MI samples (nm) (N = 69)		108	109	106	79	110	113	111	88
Correlation r(p) (Standard method 2)		0.69(<0.0001)	0.67(<0.0001)	0.61(<0.0001)	0.30(0.02)	0.68(<0.0001)	0.66(<0.0001)	0.62(<0.0001)	0.57(<0.0001)
		**T1**	**T2**	**T3**	**T4**	**T5**	**T6**	**T7**	**T8**
Histogram Mean for 69 SEM images of MI samples (nm) (N = 69)		88	81	86	79	93	86	84	84
Correlation r(p) (Standard method 2)		0.36(0.004)	0.13(0.32)	0.32(0.13)	0.51(0.0009)	0.37(0.003)	0.27(0.03)	0.16(0.21)	0.15(0.24)

Table S5. *Average arithmetic means determined by 24 algorithms included in DiameterJ and their correlations with standard manual method 2 for 69 SEM images of acute MI samples (set 2).*

	Standard method 2	M1	M2	M3	M4	M5	M6	M7	M8
Arithmetic Mean for 69 SEM images of MI samples (nm) (N = 69)	127	128	131	128	111	101	99	95	90
Correlation r* (Standard method 2)		0.76	0.76	0.72	0.71	0.62	0.59	0.53	0.70

		S1	S2	S3	S4	S5	S6	S7	S8
Arithmetic Mean for 69 SEM images of MI samples (nm) (N = 69)		121	122	119	90	123	127	124	98
Correlation r* (Standard method 2)		0.73	0.77	0.66	0.50	0.75	0.75	0.71	0.69

		T1	T2	T3	T4	T5	T6	T7	T8
Arithmetic Mean for 69 SEM images of MI samples (nm) (N = 69)		102	95	99	90	108	100	99	98
Correlation r* (Standard method 2)		0.63	0.51	0.61	0.70	0.65	0.57	0.50	0.51

***** p<0.0001 for all***

Table S6. *Average histogram means determined by 24 algorithms included in DiameterJ and their correlations with Standard manual method 3 for 150 SEM images of plasma samples (set 3).*

	Standard method 3	M1	M2	M3	M4	M5	M6	M7	M8
Histogram Mean for 150 SEM images of plasma samples (nm)(N=150)	191	237	233	226	209	229	230	214	212
Correlation r* (Standard method 3)		0.75	0.72	0.78	0.66	0.79	0.72	0.79	0.53
		**S1**	**S2**	**S3**	**S4**	**S5**	**S6**	**S7**	**S8**
Histogram Mean for 150 SEM images of plasma samples (nm)(N=150)		232	229	222	200	234	231	225	208
Correlation r* (Standard method 3)		0.75	0.72	0.80	0.67	0.75	0.75	0.79	0.70
		**T1**	**T2**	**T3**	**T4**	**T5**	**T6**	**T7**	**T8**
Histogram Mean for 150 SEM images of plasma samples (nm)(N=150)		228	214	229	210	243	225	222	221
Correlation r* (Standard method 3)		0.78	0.79	0.73	0.55	0.81	0.81	0.77	0.74

**** p<0.0001 for all***

Table S7. *Average arithmetic means determined by 24 algorithms included in DiameterJ and their correlations with Standard manual method 3 for 150 SEM images of plasma samples (set 3).*

	Standard method 3	M1	M2	M3	M4	M5	M6	M7	M8
Arithmetic Mean for 150 SEM images of plasma samples(nm)(N=150)	191	270	264	254	228	253	255	235	231
Correlation r* (Standard method 3)		0.65	0.65	0.71	0.54	0.74	0.65	0.73	0.46
		**S1**	**S2**	**S3**	**S4**	**S5**	**S6**	**S7**	**S8**
Arithmetic Mean for 150 SEM images of plasma samples (nm)(N=150)		264	260	250	216	268	264	253	226
Correlation r* (Standard method 3)		0.67	0.62	**0.72**	0.60	0.67	0.66	0.70	0.60
		**T1**	**T2**	**T3**	**T4**	**T5**	**T6**	**T7**	**T8**
Arithmetic Mean for 150 SEM images of plasma samples (nm)(N=150)		253	235	254	228	271	249	245	244
Correlation r* (Standard method 3)		0.74	0.73	0.66	0.47	0.77	0.77	0.70	0.67

**** p<0.0001 for all***

Table S8. *The correlation between diameter values determined by standard manual method 3 and automated fiber diameter measurements and biophysical properties of clots of plasma fibers and (N = 30 participants, 150 images).*

**Variable**	**Fibrinogen concentration r (p)**	**Maximum absorbance r (p)**	**Permeability (K_s) r (p)**	**Storage modulus (G') r (p)**	**Loss modulus(G")** **r (p)**	**Tan Delta (G'/G") r (p)**
**Diameter**						
- Standard manual (nm)	0.44 (0.01)	0.46 (0.01)	-0.04 (0.90)	0.27 (0.2)	0.29 (0.1)	-0.06 (0.7)
- M1 (nm)	0.28(0.14)	0.37(0.05)	0.14(0.50)	0.29(0.12)	0.27(0.14)	-0.09(0.63)
- M2 (nm)	0.28(0.13)	0.37(0.05)	0.11(0.50)	0.30(0.10)	0.29(0.12)	-0.07(0.70)
- M3 (nm)	0.33(0.07)	0.39(0.04)	0.08(0.70)	0.27(0.16)	0.26(0.17)	-0.01(0.97)
- M4 (nm)	0.33(0.08)	0.43(0.02)	-0.02(0.90)	0.25(0.18)	0.25(0.17)	0.09(0.62)
- M5 (nm)	0.33(0.08)	0.44(0.02)	0.07(0.70)	0.32(0.08)	0.27(0.16)	-0.17(0.37)
- M6 (nm)	0.26(0.17)	0.35(0.06)	0.12(0.52)	0.27(0.15)	0.20(0.27)	-0.16(0.40)
- M7 (nm)	0.30(0.11)	0.40(0.03)	0.10(0.59)	0.29(0.12)	0.25(0.17)	-0.13(0.47)
- M8 (nm)	0.51(0.01)	0.57(0.006)	-0.16(0.47)	0.30(0.18)	0.29(0.19)	-0.13(0.57)
- S1 (nm)	0.29(0.13)	0.45(0.01)	-0.02(0.93)	0.38(0.04)	0.33(0.07)	-0.18(0.33)
- S2 (nm)	0.26(0.17)	0.39(0.04)	0.03(0.87)	0.40(0.03)	0.33(0.08)	-0.15(0.43)
- S3 (nm)	0.27(0.14)	0.40(0.03)	0.11(0.57)	0.33(0.07)	0.29(0.12)	-0.14(0.45)
- S4 (nm)	0.29(0.12)	0.40(0.03)	0.06(0.74)	0.21(0.27)	0.20(0.30)	0.030(0.87)
- S5 (nm)	0.29(0.12)	0.39(0.03)	0.12(0.54)	0.28(0.13)	0.24(0.19)	-0.13(0.50)
- S6 (nm)	0.25(0.18)	0.37(0.05)	0.15(0.43)	0.34(0.07)	0.30(0.10)	-0.18(0.35)
- S7 (nm)	0.30(0.10)	0.39(0.03)	0.09(0.62)	0.32(0.08)	0.30(0.11)	-0.09(0.64)
- S8 (nm)	0.28(0.14)	0.43(0.02)	0.01(0.96)	0.28(0.13)	0.29(0.13)	0.06(0.77)
- T1 (nm)	0.33(0.08)	0.44(0.01)	0.065(0.73)	0.33(0.8)	0.27(0.15)	-0.17(0.37)
- T2 (nm)	0.30(0.11)	0.40(0.03)	0.10(0.62)	0.30(0.11)	0.26(0.17)	-0.13(0.50)
- T3 (nm)	0.23(0.23)	0.37(0.05)	0.10(0.60)	0.30(0.12)	0.24(0.21)	-0.14(0.47)
- T4 (nm)	0.58(0.003)	0.58(0.003)	-0.12(0.58)	0.23(0.27)	0.21(0.32)	-0.12(0.59)
- T5 (nm)	0.32(0.08)	0.42(0.02)	0.071(0.70)	0.34(0.07)	0.28(0.13)	-0.20(0.28)
- T6 (nm)	0.33(0.07)	0.43(0.02)	0.03(0.87)	0.28(0.14)	0.24(0.20)	-0.06(0.73)
- T7 (nm)	0.34(0.07)	0.40(0.03)	-0.002(0.99)	0.10(0.60)	0.10(0.60)	0.13(0.48)
- T8 (nm)	0.34(0.07)	0.40(0.03)	-0.02(0.93)	0.09(0.64)	0.09(0.64)	0.15(0.44)

Table S9. *The correlation between permeability (K_s) and automated porosity calculations determined by algorithms included in DiameterJ and biophysical properties of clots of plasma fibers and (N = 30 participants, 150 images).*

Variable	Fibrinogen concentration r (p)	Maximum absorbance r (p)	Permeability (K_s) r (p)	Storage modulus (G') r (p)	Loss modulus(G") r (p)	Tan Delta (G'/G") r (p)
**Porosity**						
- Permeability K_s (cm^2 x 10^{-9})	-0.54 (0.003)	-0.67 (<0.0001)	-	-0.48 (0.007)	-0.51 (0.004)	-0.18 (0.4)
- M1 Mean pore area (μm^2)	-0.22(0.24)	0.09(0.62)	0.20(0.29)	0.12(0.54)	0.15(0.41)	0.07(0.70)
- M2 Mean pore area (μm^2)	-0.08(0.68)	0.06(0.76)	0.19(0.31)	0.07(0.69)	0.13(0.50)	0.11(0.55)
- M3 Mean pore area (μm^2)	0.26(0.16)	0.35(0.06)	0.14(0.47)	0.33(0.07)	0.30(0.11)	-0.18(0.34)
- M4 Mean pore area (μm^2)	-0.00(0.10)	0.13(50)	0.22(0.24)	0.23(0.22)	0.20(0.28)	-0.18(0.33)
- M5 Mean pore area (μm^2)	-0.06(0.75)	0.13(0.51)	0.30(0.11)	0.12(0.53)	0.14(0.46)	0.03(0.87)
- M6 Mean pore area (μm^2)	-0.03(0.88)	0.24(0.20)	0.10(0.61)	0.21(0.28)	0.23(0.23)	0.06(0.77)
- M7 Mean pore area (μm^2)	0.28(0.14)	0.43(0.02)	0.09(0.65)	0.35(0.06)	0.30(0.11)	-0.17(0.36)
- M8 Mean pore area (μm^2)	-0.08(0.74)	-0.21(0.34)	0.27(0.23)	0.08(0.73)	0.03(0.88)	-0.22(0.33)
- S1 Mean pore area (μm^2)	-0.25(0.18)	0.01(0.94)	0.34(0.07)	0.15(0.44)	0.18(0.33)	-0.00(0.99)
- S2 Mean pore area (μm^2)	-0.08(0.67)	0.12(0.53)	0.16(0.41)	0.18(0.36)	0.19(0.33)	-0.05(0.81)
- S3 Mean pore area (μm^2)	0.20(0.30)	0.37(0.05)	0.14(0.47)	0.40(0.03)	0.36(0.05)	-0.25(0.19)
- S4 Mean pore area (μm^2)	-0.16(0.39)	0.08(0.68)	0.16(0.40)	0.37(0.05)	0.36(0.05)	-0.22(0.25)
- S5 Mean pore area (μm^2)	-0.27(0.16)	0.01(0.94)	0.31(0.10)	0.17(0.37)	0.19(0.32)	-0.03(0.86)
- S6 Mean pore area (μm^2)	-0.11(0.55)	0.10(0.61)	0.25(0.18)	0.21(0.25)	0.21(0.27)	-0.00(0.10)
- S7 Mean pore area (μm^2)	0.23(0.23)	0.35(0.06)	0.13(0.48)	0.34(0.06)	0.30(0.10)	-0.17(0.37)
- S8 Mean pore area (μm^2)	-0.19(0.32)	0.09(0.64)	0.13(0.50)	0.33(0.08)	0.32(0.09)	-0.11(0.58)
- T1 Mean pore area (μm^2)	-0.09(0.65)	0.12(0.53)	0.30(0.11)	0.12(0.52)	0.15(0.43)	0.04(0.83)
- T2 Mean pore area (μm^2)	0.22(0.24)	0.35(0.06)	0.17(0.37)	0.35(0.06)	0.31(0.10)	-0.21(0.27)
- T3 Mean pore area (μm^2)	0.06(0.78)	0.17(0.37)	0.14(0.46)	0.14(0.46)	0.16(0.39)	0.06(0.75)
- T4 Mean pore area (μm^2)	-0.22(0.30)	-0.33(0.12)	0.45(0.03)	0.06(0.78)	0.02(0.92)	-0.35(0.10)
- T5 Mean pore area (μm^2)	-0.13(0.49)	0.08(0.67)	0.41(0.02)	0.23(0.23)	0.22(0.24)	-0.19(0.32)
- T6 Mean pore area (μm^2)	-0.14(0.43)	0.08 (0.67)	0.37(0.03)	0.22(0.24)	0.19(0.32)	-0.15(0.17)
- T7 Mean pore area (μm^2)	-0.13(0.50)	0.12(0.54)	0.35(0.06)	0.36(0.05)	0.31(0.10)	-0.35(0.06)
- T8 Mean pore area (μm^2)	-0.10(0.60)	0.13(0.49)	0.32(0.08)	0.39(0.03)	0.35(0.06)	-0.36(0.05)
- M1 Porosity (%)	-0.25(0.18)	-0.14(0.46)	-0.15(0.44)	-0.08(0.66)	-0.05(0.79)	0.15(0.44)
- M2 Porosity (%)	-0.18(0.32)	-0.28(0.13)	0.09(0.64)	-0.27(0.15)	-0.24(0.20)	0.06(0.75)
- M3 Porosity (%)	0.05(0.81)	0.36(0.05)	0.06(0.75)	0.36(0.05)	0.31(0.10)	-0.24(21)
- M4 Porosity (%)	0.11(0.55)	0.17(0.37)	0.07(0.72)	0.45(0.01)	0.41(0.03)	-0.26(0.17)
- M5 Porosity (%)	-0.32(0.08)	-0.23(0.22)	0.19(0.32)	-0.10(0.59)	-0.01(0.94)	0.22(0.24)
- M6 Porosity (%)	-0.05(0.80)	0.1(0.62)	-0.03(0.89)	0.08(0.68)	0.13(0.49)	0.09(0.64)
- M7 Porosity (%)	0.29(0.11)	0.47(0.008)	0.00(0.96)	0.26(0.17)	0.24(0.20)	-0.16(0.93)
- M8 Porosity (%)	-0.24(0.29)	-0.35(0.10)	0.23(0.31)	-0.06(0.80)	-0.07(0.76)	-0.05(0.81)
- S1 Porosity (%)	-0.30(0.11)	-0.19(0.31)	0.18(0.36)	-0.08(0.68)	0.00(0.99)	0.17(0.38)
- S2 Porosity (%)	-0.13(0.50)	-0.13(0.50)	0.06(0.73)	-0.13(0.51)	-0.07(0.71)	0.00(0.97)
- S3 Porosity (%)	0.11(0.55)	0.40(0.03)	0.03(0.88)	0.35(0.06)	0.35(0.06)	-0.01(0.95)
- S4 Porosity (%)	-0.19(0.31)	-0.02(0.90)	-0.06(0.74)	0.31(0.10)	0.32(0.09)	-0.13(0.51)
- S5 Porosity (%)	-0.33(0.08)	-0.21(0.27)	0.04(0.82)	-0.02(0.91)	0.03(0.87)	0.09(0.64)
- S6 Porosity (%)	-0.26(0.17)	-0.28(0.13)	0.06(0.75)	-0.17(0.36)	-0.16(0.41)	0.12(0.51)
- S7 Porosity (%)	-0.10(0.57)	0.17(0.38)	0.20(0.28)	0.19(0.31)	0.22(0.24)	0.00(0.99)
- S8 Porosity (%)	-0.15(0.43)	-0.08(0.69)	0.19(0.30)	0.25(0.19)	0.20(0.28)	-0.39(0.04)
- T1 Porosity (%)	-0.33(0.7)	-0.24(0.21)	0.19(0.33)	-0.10(0.60)	-0.01(0.95)	0.23(0.23)
- T2 Porosity (%)	-0.15(0.43)	0.15(0.45)	0.29(0.12)	0.28(0.13)	0.30(0.11)	-0.15(0.44)
- T3 Porosity (%)	0.14(0.48)	0.08(0.68)	-0.01(0.96)	0.05(0.81)	0.10(0.62)	0.10(0.62)
- T4 Porosity (%)	-0.42(0.04)	-0.39(0.06)	0.33(0.12)	-0.00(0.10)	-0.02(0.91)	-0.11(0.61)
- T5 Porosity (%)	-0.50(0.004)	-0.23(0.20)	0.42(0.20)	-0.05(0.78)	-0.01(0.96)	-0.05(0.79)
- T6 Porosity (%)	-0.42(0.02)	-0.25(0.18)	0.41(0.02)	0.07(0.71)	0.08(0.67)	-0.20(0.27)
- T7 Porosity (%)	-0.24(0.21)	-0.11(0.56)	0.30(0.10)	0.29(0.13)	0.26(0.16)	-0.36(0.06)
- T8 Porosity (%)	-0.23(0.22)	-0.11(0.56)	0.31(0.10)	0.32(0.08)	0.29(0.12)	-0.39(0.03)

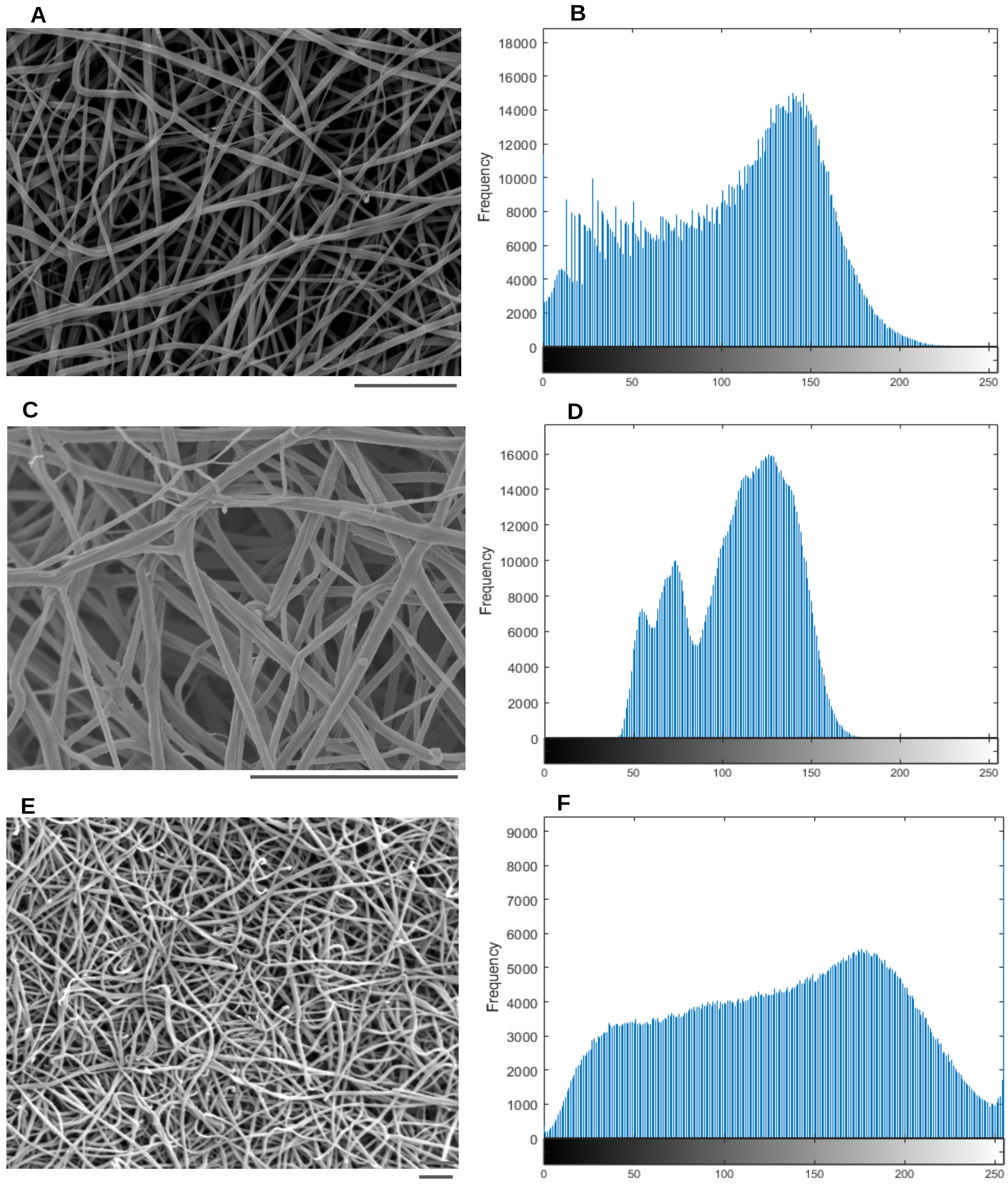

Figure S3. Representative images from (A) set 1, (B) set 2 and (C) set 3, and the corresponding distributions of gray scale levels.

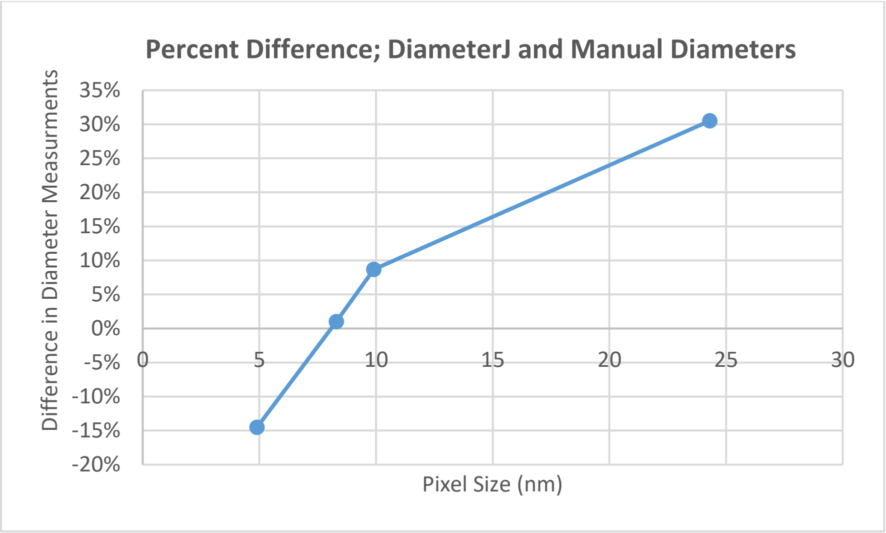

Pixel Size	Δ (Diameter)
4.9	-14.50%
8.3	1%
9.9	8.70%
24.3	30.50%

Figure S4 Difference in diameter values between DiameterJ automated measurements (arithmetic mean, average for all algorithms) and manual measurements versus pixel size.

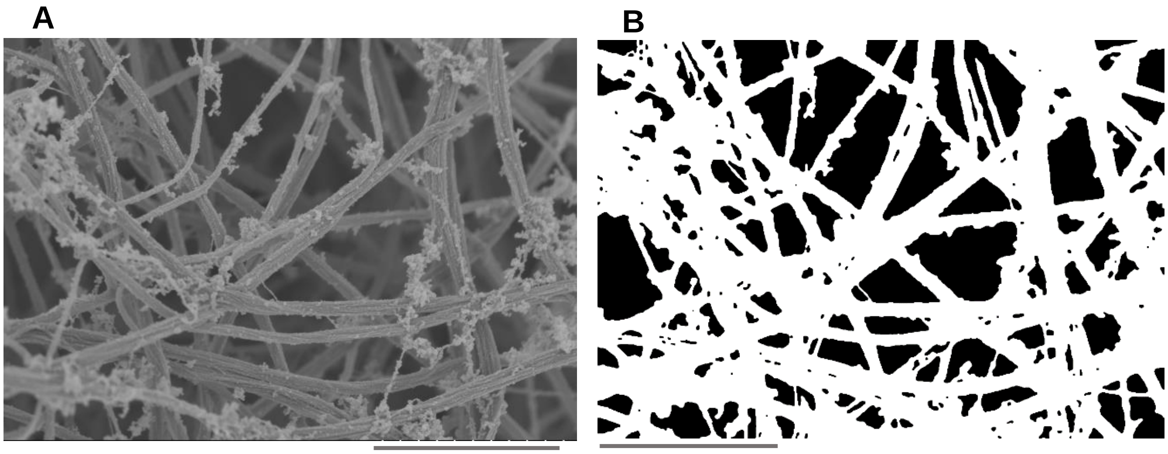

Figure S5. Particles (of unknown origin; possibly microvesicles, especially from platelets, since they have αIIbβ3 in their membranes) attached to fibrin fibers results in poor image segmentation by DiameterJ. Scale bars, 2 μm.

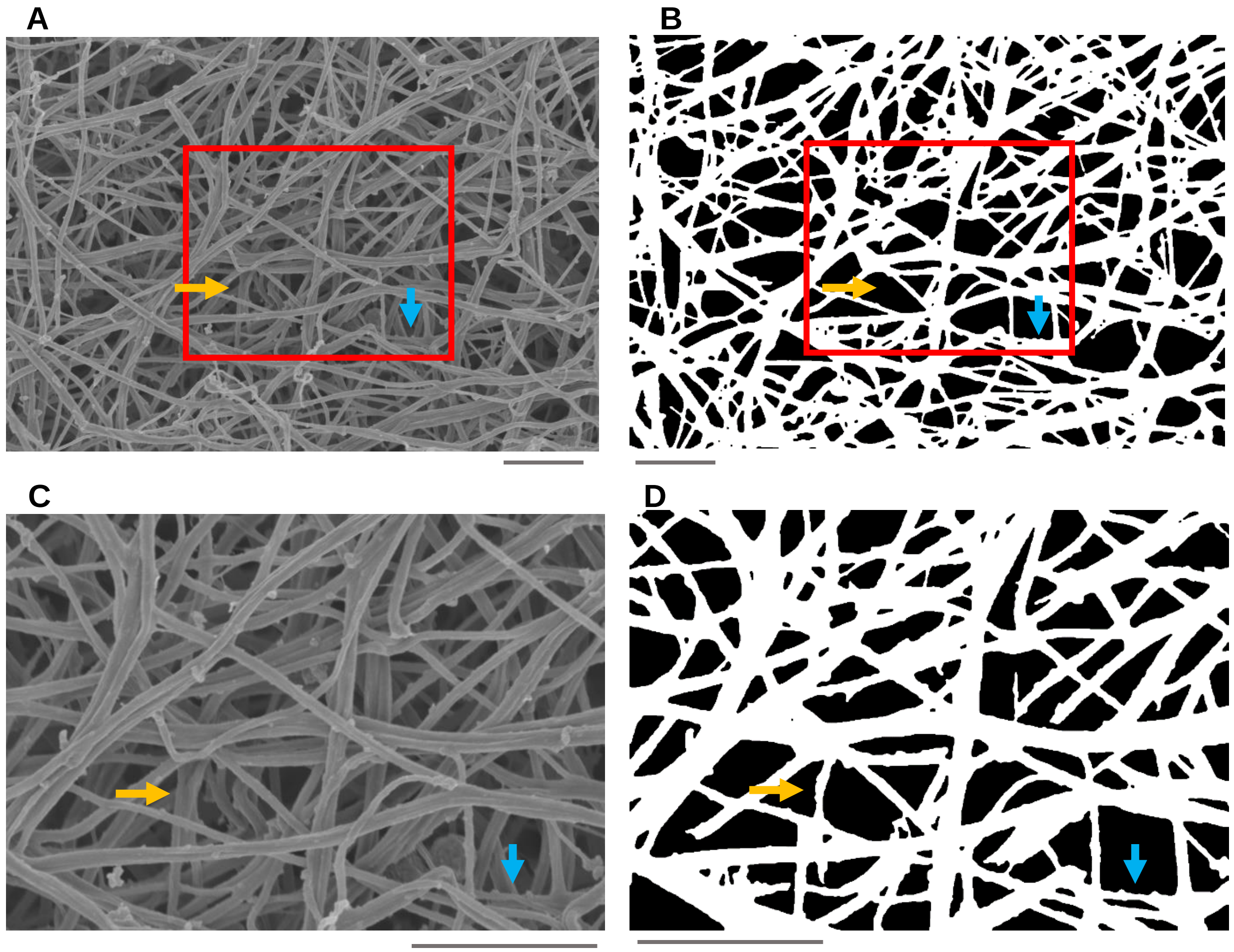

Figure S6. Mechanisms how smaller pixels reduce fiber diameter and larger pixels increase fiber diameter. (A, B) Larger pixels, 10,000x magnification, pixel size, 9.9 nm. When two fibers are laterally bundled or very close to each other, the segmentation algorithms tend to combine these two fibers into one larger fiber, thus increasing the measured fiber diameter (blue vertical arrow). This effect increases with increasing pixel size. (C, D) Smaller pixels, 20,000x magnification, pixel size, 4.9 nm. When processing images with smaller pixel sizes, segmentation algorithms tend to include fibers that are deeper in the clot but render them as thin, incomplete fibers. This phenomenon is more prevalent in images with the smaller pixel size (yellow horizontal arrow), thus decreasing the measured diameter for these images.

References

[1] R. Nock, F. Nielsen, Statistical region merging, IEEE Trans. Pattern Anal. Mach. Intell. 26 (2004) 1452–1458. https://doi.org/10.1109/TPAMI.2004.110.
[2] C.H. Li, C.K. Lee, Minimum cross entropy thresholding, Pattern Recognit. 26 (1993) 617–625. https://doi.org/10.1016/0031-3203(93)90115-D.
[3] C.H. Li, P.K.S. Tam, An iterative algorithm for minimum cross entropy thresholding, Pattern Recognit. Lett. 19 (1998) 771–776. https://doi.org/10.1016/S0167-8655(98)00057-9.
[4] J. Kittler, J. Illingworth, Minimum error thresholding, Pattern Recognit. 19 (1986) 41–47. https://doi.org/10.1016/0031-3203(86)90030-0.
[5] N. Otsu, A Tlreshold Selection Method from Gray-Level Histograms, (n.d.) 5.
[6] F. Samopa, A. Asano, Hybrid Image Thresholding Method using Edge Detection, (2008) 8.
[7] G.W. Zack, W.E. Rogers, S.A. Latt, Automatic measurement of sister chromatid exchange frequency., J. Histochem. Cytochem. 25 (1977) 741–753. https://doi.org/10.1177/25.7.70454.
[8] L.-K. Huang, M.-J.J. Wang, Image thresholding by minimizing the measures of fuzziness, Pattern Recognit. 28 (1995) 41–51. https://doi.org/10.1016/0031-3203(94)E0043-K.
